# Exploring the Effectiveness of Imipenem/Relebactam in Patients with Antimicrobial-Resistant Hospital-Acquired Infections: Findings from Systematic Literature Reviews

**DOI:** 10.3390/antibiotics15020170

**Published:** 2026-02-05

**Authors:** Ryan K. Shields, Ignacio Martin-Loeches, Emre Yücel, Shalini Bagga, Vaneet Pal Kaur Khurana, Prashant Soni, Prateek Das, Carolyn Cameron

**Affiliations:** 1 Department of Medicine, University of Pittsburgh, Pittsburgh, PA 15260, USA; 2 Department of Intensive Care Medicine, Multidisciplinary Intensive Care Research Organization (MICRO), St. James’s Hospital, D08 NHY1 Dublin, Ireland; 3 School of Medicine, Trinity College Dublin, College Green, D02 R590 Dublin, Ireland; 4 Merck & Co., Inc., Rahway, NJ 02115, USA; 5 CHEORS LLC, Chalfont, PA 18914, USA; 6 MSD, Sydney 2113, Australia

**Keywords:** imipenem/relebactam, mortality, clinical success, microbiological success, complicated urinary tract infections, complicated intra-abdominal infections, hospital-acquired bacterial pneumonia, ventilator-associated bacterial pneumonia

## Abstract

**Introduction:** Infections attributed to multidrug-resistant organisms have resulted in a significant clinical burden, high mortality, and excessive costs. Identifying the most appropriate and efficacious treatments will aid in reducing these burdens. Imipenem/cilastatin + relebactam (I/R) is used against multidrug-resistant infections providing an alternative option which may support patients where traditional treatments are no longer effective. **Objective:** The objective was to evaluate the efficacy of I/R for complicated urinary tract infections, complicated intra-abdominal infections, hospital-acquired bacterial pneumonia, and ventilator-associated bacterial pneumonia, based on data aggregated from randomized controlled trials. **Method:** Two systematic literature reviews were conducted to include randomized controlled trials which aligned with the inclusion criteria reporting on the efficacy of I/R against placebo or other comparators such as piperacillin/tazobactam or colistin. The outcomes of interest were mortality, clinical response, and microbiological response. **Results:** The results found reduced mortality and comparable clinical and microbiological response with I/R versus its comparators. I/R displayed the largest favorable clinical and microbiological responses within high-risk populations, including those with severe renal impairment when compared with piperacillin/tazobactam. **Conclusions:** These findings support the efficacy of I/R for key Gram-negative infections, particularly within vulnerable patient populations. Despite the favorable outcomes reported, there is a need for further real-world evidence generation to support the efficacy of I/R to aid in standardizing treatment guidelines and reducing the clinical and economic burden associated with multidrug-resistant bacterial infections.

## 1. Introduction

The World Health Organization (WHO) has classified antimicrobial resistance as a global and public health concern [1]. Infections caused by multidrug-resistant (MDR) organisms have risen, leading to an estimated global mortality of 1.27 million in 2019 [2]. Once-reliable treatments for common infections are increasingly failing, leaving clinicians with fewer, less effective, and often more toxic therapeutic options. This growing crisis is driving severe clinical consequences, escalating patient suffering, and imposing crippling economic and healthcare system burdens [2].

Gram-negative bacteria such as *Escherichia coli*, *Klebsiella pneumoniae*, *Pseudomonas aeruginosa*, *Stenotrophomonas maltophilia,* and *Acinetobacter baumannii*, along with Gram-positive bacteria such as *Staphylococcus aureus* and *Streptococcus pneumoniae*, are prominently implicated in MDR infections [3,4]. Six of these pathogens, included within the WHO priority pathogens list in 2024, lead to high mortality rates and accounted for ~929,000 of 1.27 million deaths due to MDR in 2019 [3,5]. The annual mortality rate related to MDR infections is expected to increase to 10 million by 2050, emphasizing the need for new and effective treatments [6].

The emergence of MDR places increasing strain on healthcare systems due to additional healthcare resource utilization and associated costs in caring for patients [2]. Compared to non-resistant infections, Carbapenem-resistant Enterobacterales (CRE) infections can increase patients’ length of stay (LOS) by up to eight days, while MDR infections can lead to an increased LOS of more than 10 days [7,8]. Estimates from the 2014 United Kingdom (UK) O’Neill Review predicted that MDR could reduce global gross domestic product (GDP) by 2–3.5% by 2050, costing up to US $100 trillion [9]. Therefore, novel treatments are required to minimize the clinical and economic impacts of MDR infections.

MDR organisms are a major cause of hospital-acquired infections (HAIs). Transmission of MDR organisms occurs through the environment, patients, and healthcare workers [10]. Estimates suggest that nearly 8% of hospitalized patients develop an HAI, and 20% of those may be caused by MDR organisms [11]. HAI rate can reach up to 40% within intensive care units with the most common forms associated with blood stream infections, surgical site infections, and respiratory tract infections [12]. Methicillin-resistant *S. aureus* (MRSA), vancomycin-resistant *E. faecium,* and CRE are commonly reported MDR organisms associated with HAIs contributing to the severity and reduced treatability of infections in hospitals [12]. In the US, each CRE infection costs US $22,000–66,000 while MDR pathogens such as *P. aeruginosa* cost approximately US $492 million per year for HAI [13,14].

In addition to the high morbidity and mortality associated with MDR infections, there is additional risk for immunocompromised patients such as those with cancer who are particularly susceptible to acquiring MDR infections [15]. For instance, cancer patients are approximately 2.9 times more likely to decease due to an infection compared to the general population, with a 62% mortality rate attributed to sepsis [16,17]. The rise in MDR further exacerbates this vulnerability, making treatment more complicated and increasing the potential for fatal outcomes. Research has shown that MDR and extensively drug-resistant (XDR) infections significantly contribute to mortality in cancer patients, reporting 30-day mortality rates of 38.92% and 50.29%, respectively [18].

The current treatments available to combat MDR infections include β-lactam/β-lactamase inhibitor (BL/BLI) combinations such as piperacillin/tazobactam (PIP/TAZ), ceftazidime/avibactam (C/A), ceftolozane/tazobactam (C/T), meropenem/vaborbactam (MER/VAB), and imipenem/cilastatin + relebactam (I/R) [19,20,21,22,23,24,25]. These agents restore activity against serine β-lactamase producers and displace nephrotoxic polymyxin-based regimens [19]. Carbapenem/BLI agents shield carbapenems from class A and C β-lactamases. They show rapid bactericidal activity, good lung penetration, and less toxicity than older agents such as colistin and polymyxin B [26,27]. Gram-negative MDR infections rival *Mycobacterium tuberculosis* (TB) and human immunodeficiency virus (HIV) in mortality and impose huge costs. Carbapenem/β-lactamase inhibitors, especially I/R, offer broad coverage, reduced toxicity, and resistance containment [28]. I/R has been approved by the Food and Drug Administration (FDA) for complicated urinary tract infections (cUTI) (including pyelonephritis), complicated intra-abdominal infections (cIAI) in adults, and hospital-acquired bacterial pneumonia/ventilator-associated bacterial pneumonia (HABP/VABP) [29]. Additionally, I/R is an alternative for individuals with complicated infections and limited treatment options [29].

The objective of the two systematic literature reviews presented in this paper was to assess the clinical evidence from randomized controlled trials comparing I/R to alternative treatments in key serious infections—specifically cUTI (including pyelonephritis), cIAI, and HABP/VABP. The goal was to understand the role of I/R in the management of these challenging, often drug-resistant infections.

## 2. Results

### 2.1. Overview of Included Publications

#### 2.1.1. cUTI and cIAI Populations

A total of 9737 records were identified in the systematic literature review investigating the efficacy of I/R in adult patients with cUTI and cIAI.

**Figure 1:**
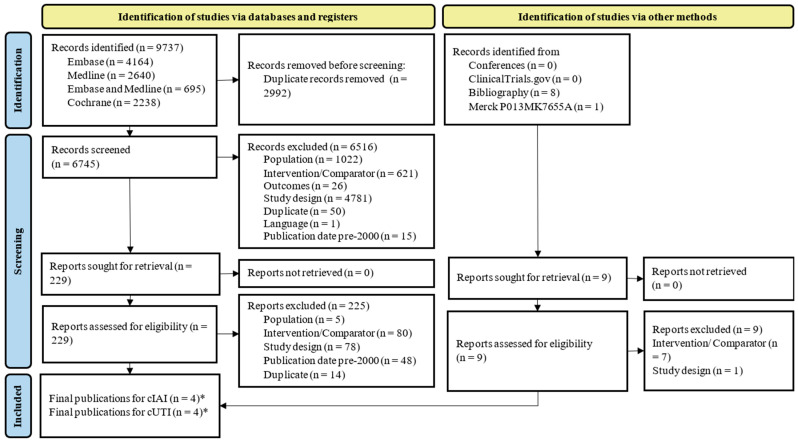
Flow diagram of study selection for cUTI and cIAI according to PRISMA guidelines. * cUTI and cIAI were both reported upon in three publications, one publication reported solely on cUTI, and one publication reported solely on cIAI; therefore, there are four publications which report on cIAI and four publications which report on cUTI. In both cUTI and cIAI systematic literature reviews, three of the included publications reported data for cUTI, cIAI, and HABP/VABP.

After title/abstract and full-text screening, five publications from three unique trials were included (Figure 1). Three of the five publications were linked to the RESTORE-IMI 1 trial which was conducted in hospitalized adult patients with Gram-negative infections such as cUTI, cIAI, and HABP/VABP. The secondary publications explored key subgroups or analyses (Table 1). Additionally, one phase II, dose-ranging study was included in each cUTI and cIAI patients. Four of the five publications investigated the outcomes of interest and were included within this review.

#### 2.1.2. HABP/VABP Populations

**Figure 2:**
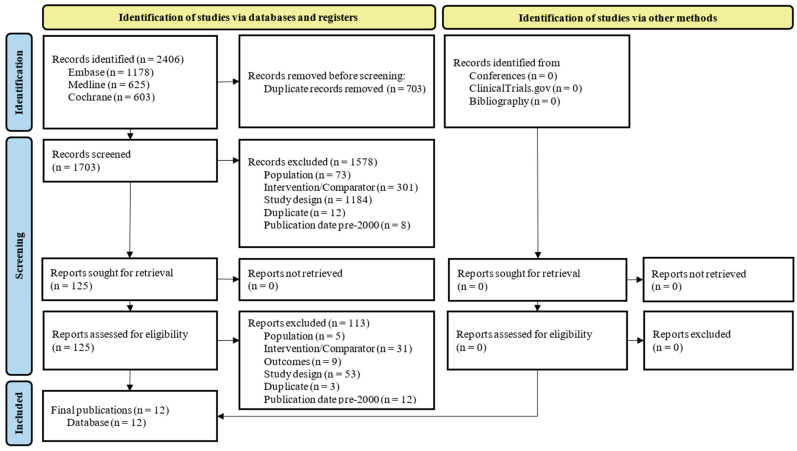
Flow diagram of study selection according to PRISMA guidelines—HABP/VABP.

In the systematic literature review on HABP/VABP, a total of 2406 database records were identified. After title/abstract and full-text screening, 12 publications were included (Figure 2). These encompassed the following three unique trials: RESTORE-IMI 1, RESTORE-IMI 2, and NCT03583333, with most publications reporting results of the RESTORE-IMI 2 trial (N = 7). A total of five of the identified publications reported on the outcomes of interest. The secondary publications of each trial (outlined in Table 2) investigated key subgroups or analyses.

### 2.2. Mortality

#### 2.2.1. Mixed Infection Types (cUTI, cIAI, and HABP/VABP)

A total of two publications reported mortality in cUTI, cIAI, and HABP/VABP populations comparing I/R with colistin + imipenem (Table 3). Both publications displayed data from the RESTORE-IMI 1 trial and reported a lower ACM on day 28 when I/R was administered compared to colistin + imipenem/cilastatin (9.5% vs. 30% and 10.7% vs. 23.1%, respectively) [28,30]. Each publication reported on microbiological modified intent-to-treat (mMITT) and supplemental microbiological modified intent-to-treat populations (SmMITT), respectively. RESTORE-IMI 1 defined the mMITT population as patients with at least one dose of the study treatment with a qualifying baseline pathogen such as *P. aeruginosa*, *Klebsiella* spp., or other *Enterobacteriaceae.*

#### 2.2.2. HABP/VABP Populations

A total of six publications reported mortality for HABP/VABP populations, comparing I/R to PIP/TAZ (Table 4). Overall, mortality was lower with I/R with five publications reporting decreased all-cause mortality on day 28 when I/R was administered compared to PIP/TAZ. A total of three publications compared mortality in the modified intent-to-treat (MITT) population, two of which reported an improvement in mortality rate when I/R was administered compared to PIP/TAZ (15.9% versus 21.3% and 14.8% versus 19.5%) [34,40]. However, one publication reported increased all-cause mortality with I/R compared to PIP/TAZ (11.2% vs. 5.9%, respectively) [42]. The RESTORE-IMI 2 trial defined the MITT patient population as patients who received the study therapy, without any Gram-positive cocci at baseline measurements. The NCT03583333 trial defined MITT as patients who received one or more doses of the study therapy without any Gram-positive cocci at baseline measurements.

In a subgroup analysis investigating patients with positive extended-spectrum beta-lactamase (ESBL), I/R administration led to 20% mortality, while PIP/TAZ administration led to 22.9% mortality at day 28 [38].

In a publication reporting ACM according to renal function, I/R showed decreased mortality compared to PIP/TAZ within the augmented renal clearance (ARC) group (7.7% versus 14.6%) [35]. Similarly, a publication investigating intensive care unit (ICU) patients displayed decreased mortality when I/R was administered compared to PIP/TAZ (17.1% versus 23.9%) [36].

### 2.3. Clinical Response

#### 2.3.1. cUTI and cIAI Populations

A total of two publications reported on clinical response in combined population of patients with cUTI, cIAI, and HABP/VABP, comparing I/R with colistin + imipenem (Table 5). Each publication reported on mMITT and SmMITT. One publication reported clinical response in patients with cUTI only comparing I/R at two different doses (125 mg and 250 mg) to placebo investigating the microbiological evaluable (ME) population (Table 6).

Both publications comparing I/R to colistin + imipenem were based on the RESTORE-IMI 1 trial and reported a more favorable clinical response when I/R was administered at different time points of outcome collection or at day 28 [28,30]. The publication reporting on NCT01505634 within the cUTI population found a similar clinical response between both doses of I/R and placebo [32]. Clinical response was favorable in both Gram-negative aerobic and anaerobic bacilli and Gram-positive aerobic cocci [33].

#### 2.3.2. HABP/VABP Populations

A total of seven publications reported on clinical response with I/R compared to PIP/TAZ in patients with HABP/VABP (Table 7). These publications reported results from RESTORE-IMI 1, RESTORE-IMI 2, and NCT03583333 reporting on MITT and subgroup populations.

Variations were observed depending on the timepoint of data collection and which subpopulation was analyzed; however, all results presented favorable clinical response for I/R when compared to PIP/TAZ. Notably, in the publication by Roberts (2023) investigating the efficacy of I/R among HABP/VABP patients with differentiating renal function from the RESTORE-IMI 2 trial, favorable clinical response was greatest in patients with an ARC ≥250 when compared to normal renal function (NRF) (91.7% vs. 44.4% and 66.0% vs. 61.2%, respectively) [35]. Favorable clinical response due to I/R when compared to PIP/TAZ was also demonstrated in ICU patients (58.9% vs. 54.5%) [36].

### 2.4. Microbiological Response

#### 2.4.1. cUTI and cIAI Populations

Two publications reported microbiological outcomes at different time points since treatment initiation based on NCT01505634 and NCT01506271 (Table 8). I/R 125 mg and 250 mg were compared to imipenem/cilastatin + placebo in both publications. Both publications reported similar or improved microbiological outcomes at late follow-up; however, one publication found that favorable microbiological response was higher at early follow-up with imipenem/cilastatin + placebo [32,33]. The ME population was defined as patients who met the protocol definitions of cUTI or cIAI, with growth of at least one Gram-negative pathogen, and received greater than 96 h of the study therapy in both NCT01505634 and NCT01506271.

#### 2.4.2. HABP/VABP Populations

Five publications reported on microbiological response for HABP/VABP populations from the RESTORE-IMI 2 and NCT03583333 trials investigating the MITT, ME, and subgroup populations (Table 9).

Most publications reported similar or favorable microbiologic response with I/R compared to PIP/TAZ. The largest benefit in microbiological response was observed in patients administered with I/R with severe renal impairment (RI) (57.1% versus 42.9%) [35].

### 2.5. Risk of Bias

#### 2.5.1. cUTI and cIAI Systematic Literature Review

Overall, the three trials included in the systematic literature review had low risk of bias for the domains pertaining to selection bias (random sequence generation sub-domain), performance bias, detection bias, attrition bias, and reporting bias. The sub-domain of allocation concealment within the selection bias domain had an unclear risk in all studies. The risk of other bias was found to be high in two studies, while one study had an unclear risk. These results are outlined in Figure S1.

#### 2.5.2. HABP/VABP Systematic Literature Review

Overall, the three trials included in the systematic literature review had low risk of bias for the domains pertaining to selection bias (random sequence generation sub-domain), performance bias, detection bias, attrition bias, and reporting bias. The sub-domain of allocation concealment within the selection bias domain had an unclear risk in all the studies. The risk of other bias was found to be high in two studies, while one study had an unclear risk. These results are outlined in Figure S2.

## 3. Discussion

This systematic literature review evaluated the efficacy of imipenem/relebactam (I/R) across the following three serious infection types: complicated urinary tract infections (cUTIs), complicated intra-abdominal infections (cIAIs), and hospital-acquired/ventilator-associated bacterial pneumonia (HABP/VABP). Overall, the findings support the clinical utility of I/R as an effective treatment option for adult patients with these infections, including those caused by MDR Gram-negative pathogens. Across publications, I/R demonstrated comparable or improved clinical and microbiological response rates, and in several trials, notably lower mortality rates versus comparators such as colistin or piperacillin/tazobactam. These results are particularly relevant in the context of rising antimicrobial resistance, where safe and effective alternatives to older, more toxic agents are urgently needed.

Overall, most publications (N = 7) reported decreased mortality with I/R versus colistin + imipenem/cilastatin or PIP/TAZ across all populations. Sub-group analysis of data from RESTORE-IMI 2 reported lower all-cause mortality on day 28 for patients with HABP/VABP who were in the ICU and received I/R (17.1%) compared to PIP/TAZ (21.3%), demonstrating the efficacy of I/R in critically ill patients. Furthermore, in this trial, patients with both normal renal function and reduced augmented renal clearance (ARC ≥ 150) experienced reduced all-cause mortality with I/R, suggesting that I/R provides consistent efficacy across different levels of renal function. In RESTORE-IMI 1, the I/R cohort displayed reduced mortality compared to the colistin + imipenem group (9.5% versus 30%) when considering the total population (patients with cUTI, cIAI, and HABP/VABP). These results are of particular importance as the study population in RESTORE-IMI 1 consisted of MDR patients with high APACHE II scores and comorbidities, leading to increased treatment complexity and higher risk of mortality, therefore demonstrating the efficacy of I/R in high-risk populations.

Overall, I/R demonstrated similar or more favorable clinical outcomes across cUTI, cIAI, and HABP/VABP populations versus comparators. In RESTORE-IMI 1, I/R led to a more favorable clinical response compared to colistin + imipenem in patients with cUTI, cIAI, and HABP/VABP. These results align with a systematic literature review conducted by Sansone et al. (2024) [43] which aimed to summarize publications investigating the use of I/R in patients with MDR Gram-negative infections. They found that 72.9% and 72.7% of patients achieved a similar clinical response with I/R and colistin + imipenem, respectively [43]. In RESTORE-IMI 1, sub-group analysis of the cUTI population demonstrated a similar favorable clinical response in the I/R group compared to the colistin + imipenem group at both EFU and LFU. These results are comparable to a publication by Sims et al. (2017) which demonstrated that I/R was equally as effective as imipenem/cilastatin for treating cUTI, showing favorable clinical outcomes across all treatment groups, including those with MDR strains [32]. RESTORE-IMI 2 confirmed non-inferiority of I/R to PIP/TAZ in HABP/VABP when considering clinical response [34]. Subgroup analyses in vulnerable patient populations displayed increased clinical outcomes when I/R was administered when compared to PIP/TAZ, as seen by the difference in patients with ARC and NRF (91.7% vs. 44.4% and 66.0% vs. 61.2% respectively), therefore demonstrating where I/R is best applied.

Microbiological responses from RESTORE-IMI 2, NCT03583333, NCT01505634, and NCT01506271 were similar when comparing I/R 125 mg and 250 mg to imipenem/cilastatin + placebo in cUTI/cIAI populations. These results are comparable to a review conducted by Heo et al. (2021) which reported non-inferiority of I/R compared to imipenem/cilastatin + placebo [44]. Findings from publications in the HABP/VABP population displayed a favorable microbiological response, especially in high-risk patients. The greatest improvement in microbiological response was reported from RESTORE-IMI 2 in patients with severe RI comparing I/R with PIP/TAZ (57.1% versus 42.9%), further emphasizing the efficacy of I/R in vulnerable patients. Importantly, I/R also demonstrated robust activity against CRE and multidrug-resistant Pseudomonas aeruginosa, highlighting its value as a potent antipseudomonal agent in difficult-to-treat infections. This paper consolidates evidence to support clinical decision making on the use of I/R for MDR infections. There has been a significant shift in the treatment of infections away from polymyxin-based therapies and toward novel BL/BLI combinations such as I/R. This change is largely driven by the considerable advantage of reduced nephrotoxicity offered by the newer agents and improved management of MDR infections. To ensure the longevity of I/R, it is important to implement strong antimicrobial stewardship programs that focus on appropriate use. This includes ongoing surveillance for the emergence of resistance and the development of strategies to mitigate its spread.

## 4. Materials and Methods

Two systematic literature reviews were conducted. One systematic literature review was focused on randomized controlled trials related to cUTI and cIAI indications, while the second systematic literature review was conducted to evaluate the efficacy of I/R in adult patients with HABP/VABP. As the systematic literature reviews were conducted in separate populations, there was no risk of overlap so measures to avoid double-counting or over-weighting of individual trials in the qualitative synthesis were not warranted. Both systematic literature reviews were conducted in accordance with the Preferred Reporting Items for Systematic Reviews and Meta-Analyses (PRISMA) guidelines to improve transparency and reproducibility. Non-English studies were excluded. Key MeSH descriptors included within the cUTI and cIAI systematic literature reviews were ‘Urinary Tract Infections’, Pyelonephritis’, ‘Catheter-Related Infections’, and ‘Intra-Abdominal Infection’ while key search terms for HABP/VABP included ‘Respiratory Tract Infection’ and ‘Pneumonia’. The completed PRISMA checklist is provided in the Supplementary Materials.

Inclusion criteria for both systematic literature reviews were based on the PICOTS framework to ensure clarity and consistency in evidence synthesis (Table S1). The PICOTS inclusion criteria included adults with a bacterial infection causing cUTI, cIAI, or HABP/VABP being treated with I/R, compared to another combination, in randomized controlled trials. The outcomes of interest included ACM, clinical response, and microbiological response.

Systematic searches were conducted in PubMed and MEDLINE, EMBASE, and Cochrane databases, from inception up to 15 July 2024. Relevant conference proceedings from the last two years and bibliographies of identified systematic reviews were also hand-searched (Tables S2–S25).

Abstract and full-text screening were conducted by two independent reviewers based on the PICOTS criteria and any conflicts were resolved by a third senior reviewer. Data extraction was performed using a predefined template by one reviewer and checked for accuracy by the second reviewer. The quality of included publications was assessed using the revised Cochrane risk of bias tool.

The definition of favorable clinical response was similar across all randomized controlled trials included in the systematic literature reviews, defined as the alleviations of symptoms and signs from baseline. Comparably, favorable microbiological response was defined as the eradication of baseline pathogens across all randomized controlled trials. Each definition included slight variations to these. In the instance that a publication was included within both systematic literature reviews, the results were deduplicated and added within tables indicating relevance in cIAI, cUTI, and HABP/VABP populations.

## 5. Conclusions

The systematic literature reviews presented in this paper provide compelling evidence supporting the efficacy of I/R for the treatment of cUTI, cIAI, and HABP/VABP, particularly in critically ill patients and those with impaired renal function, as demonstrated in the RESTORE-IMI 1 and RESTORE-IMI 2 trials. I/R appears to be a valuable therapeutic option in settings where pathogens exhibit resistance to standard treatments, offering a potential solution to the growing challenge of antimicrobial resistance AMR. These findings may justify the broader incorporation of I/R into clinical practice and treatment guidelines across diverse geographic regions. Nonetheless, further research is needed to validate the efficacy and safety of I/R across wider patient populations and infection types, with greater sample sizes, across more subgroup populations including more diverse populations globally, and with longer follow-up periods, to support consistent clinical decision making and reduce the global burden of MDR-related morbidity, mortality, and healthcare costs.

## 6. Limitations

These systematic literature reviews are subject to several limitations that should be acknowledged. First, inherent biases within the included publications may influence the overall interpretation of the findings. Differences in study design, patient populations, and outcome definitions may have introduced heterogeneity that affects comparability across trials. Additionally, variation in how outcomes were reported, particularly regarding clinical and microbiological response, may have impacted the consistency and reliability of the aggregated data. These systematic literature reviews had a small number of included publications which could be attributed to the limitations set out within the PICOTS criteria which required I/R to be an intervention investigated against the four indications within a clinical trial. Adherence to the PICOTS criteria resulted in a small number of included publications but was essential for the systematic nature of this review. This review aims to consolidate the findings of I/R in these specific clinical settings in order to inform further clinical decision making. In addition, selection bias may have been introduced during the hand searches of conferences and trials. In order to avoid these biases, there was strict adherence to PICOT criteria outlined in the methodology. Furthermore, a RoB assessment was conducted for each included article to ascertain the level of bias which was concluded to be low overall (Figures S1 and S2). As this review was sponsored, the risk of additional bias increases; however, the systematic nature of the review and the adherence to PRISMA guidelines reduces this sponsorship bias. Subgroup findings are exploratory and underpowered due to the limited data from this systematic literature review. Another important limitation is that most of the randomized controlled trials included were designed as non-inferiority trials. While appropriate for regulatory approval, these trials are not intended to demonstrate superiority and therefore do not provide definitive insight into whether I/R offers significant clinical advantage over standard-of-care comparators. Finally, there remains a significant gap in the availability of head-to-head randomized controlled trials evaluating I/R versus alternative agents in well-defined, real-world populations. This underscores the need for robust real-world evidence to complement clinical trial data, guide optimal antimicrobial selection, and support the development of standardized treatment pathways.

## Figures and Tables

**Table 1:** Basic characteristics of each publication reporting outcomes of interest—cUTI and cIAI.

Publication	Primary or Secondary Publication	Patient Enrollment Time	No. of Sites/Centers	Study Design	Selection Criteria for Patients
**cUTI, cIAI and HABP/VABP (RESTORE-IMI 1; NCT02452047)**					
**Motsch (2020)** [28]	Primary	October 2015–September 2017	35 hospitals in 17 countries	Phase 3, double-blind, randomized controlled trial	Hospitalized adult patients with cUTI, cIAI, and HABP/VABP infections caused by imipenem-resistant Gram-negative bacteria who require IV antibiotics
**Kaye (2020)** [30]	Secondary	October 2015–September 2017	35 hospitals in 17 countries		
Brown (2020) * [31]	Secondary	October 2015–September 2017	16 sites in 11 countries		
**cUTI (NCT01505634)**					
**Sims (2017)** [32]	Primary	December 2012–July 2015	34 hospitals in 11 countries	Phase 2b, double-blind, randomized controlled trial	Hospitalized adult patients with documented cUTI or acute pyelonephritis infections who require IV antibiotics
**cIAI (NCT01506271)**					
**Lucasti (2016)** [33]	Primary	16 November 2012–12 September 2014	45 sites in 20 countries	Phase 2, double-blind, randomized controlled trial	Hospitalized adult patients with documented cIAI infections who require IV intravenous antibacterial therapy

Abbreviations: cIAI: complicated intra-abdominal infection; cUTI: complicated urinary tract infection; IV: intravenous; HABP: hospital-acquired pneumonia; and VABP: ventilator-associated pneumonia. * this publication was not included within the results as it did not report outcomes of interest for this analysis.

**Table 2:** Basic characteristics of each publication reporting outcomes of interest—HABP/VABP.

Publication	Primary or Secondary Publication	Patient Enrollment Time	No. of Sites/Centers	Study Design	Selection Criteria for Patients
**HABP/VABP (RESTORE-IMI 2; NCT02493764)**					
**Titov (2021)** [34]	Primary	January 2016–April 2019	113 hospitals in 27 countries	Phase 3, double-blind, active comparator, randomized controlled trial	Adult patients who require iv antibiotics for non-ventilated HABP, ventilated HABP, or VABP, with a baseline LRT specimen collected within 48 h of screening and meeting clinical, radiographic, and laboratory criteria for pneumonia
**Roberts (2023)** [35]	Secondary	November 2015–April 2019			
**Chen (2020)** [36]	Secondary				
**Martin-Loeches (2023)** [37] *	Secondary				
**Losada (2020A)** [38]	Secondary				
**Losada (2020B)** [39] *	Secondary				
**EUCTR Registry (2018)** [40]	Secondary				
**HABP/VABP (NCT03583333)**					
**Clinicaltrial.gov registry (2018)** [41] *	Primary	September 2018–July 2022	54 sites in eight countries	Phase 3, double-blind, active comparator, randomized controlled trial	Adult patients who require iv antibiotics for HABP or VABP, with a baseline LRT specimen collected and who met clinical and radiographic criteria for pneumonia with onset of symptoms after more than 2 days of hospitalization, within 7 days of discharge, or after mechanical ventilation
**Li (2023)** [42]	Secondary				
**cIAI, cUTI and HABP/VABP (RESTORE-IMI 1; NCT02452047)**					
**Motsch (2020)** [28]	Primary	October 2015–September 2017	35 hospitals in 17 countries	Phase 3, double-blind, active comparator, randomized controlled trial	Hospitalized adult patients with cUTI, cIAI, and HABP/VABP infections caused by imipenem-resistant Gram-negative bacteria who require IV antibiotics
**Kaye (2020)** [30]	Secondary				
**Brown (2020)** [31] *	Secondary				

Abbreviations: cIAI: complicated intra-abdominal infection; cUTI: complicated urinary tract infection; HABP: hospital-acquired bacterial pneumonia; IV: intravenous; LRT: lower respiratory tract; and VABP: ventilator-associated bacterial pneumonia. * these publications were not included within the results as they did not report outcomes of interest for this analysis.

**Table 3:** Mortality of cUTI, cIAI, and HABP/VABP populations.

Publication	Outcome	I/R % (n/N)	Colistin + Imipenem/Cilastatin % (n/N)	Difference % (90% CI)
**RESTORE-IMI 1**				
**cUTI, cIAI, and HABP/VABP (mMITT)**				
**Motsch (2020)** [28]	All-cause mortality (through day 28)	9.5% (2/21)	30.0% (3/10)	−17.3 (−46.4 to 6.7)
**cUTI, cIAI, and HABP/VABP (SmMITT)**				
**Kaye (2020)** [30]	All-cause mortality (through day 28)	10.7% (3/28)	23.1% (3/13)	−10.5 (−35.2 to 9.6)

Abbreviations: CI: confidence interval; cIAI: complicated intra-abdominal infection; cUTI: complicated urinary tract infection; HABP: hospital-acquired pneumonia; mMITT: microbiological modified intent-to-treat; SmMITT: supplemental microbiological modified intent-to-treat; and VABP: ventilator-associated pneumonia.

**Table 4:** Mortality of HABP/VABP populations.

Publication	Outcome	I/R % (n/N)	Piperacillin + Tazobactam % (n/N)	Difference % (95 CI)
**RESTORE-IMI 2**				
**HABP/VABP (MITT)**				
**Titov (2021)** [34]	All-cause mortality on day 28	15.9% (42/264)	21.3% (57/267)	−5.3 (−11.9 to 1.2)
**HABP/VABP (subgroups by renal function)**				
**Roberts (2023)** [35]	All-cause mortality on day 28 among patients with NRF	7.8% (NA/103)	15.3% (NA/85)	−7.5 (−17.6 to 1.6)
	All-cause mortality on day 28 among patients with ARC ≥ 150 to <250	7.7% (NA/26)	14.6% (NA/41)	−6.9 (−22.5 to 11.4)
**HABP/VABP (subgroups by ICU admitted patients)**				
**Chen (2020)** [36]	All-cause mortality on day 28 for patients in ICU	17.1% (30/175)	23.9% (42/176)	−6.7 (−15.2 to 1.8)
**HABP/VABP (MITT)**				
**EUCTR Trial Registry (2018)** [40]	All-cause mortality on EFU	14.8% (NA/264)	19.5% (NA/267)	−4.6 (−11.0 to 1.7)
**HABP/VABP (subgroups by pathogens)**				
**Losada (2020)** [38]	All-cause mortality on day 28 among patients with ESBL positive	20.0% (NA/45)	22.9% (NA/35)	NA
**NCT03583333**				
**HABP/VABP (MITT)**				
**Li (2023)** [42]	All-cause mortality on day 28	11.2% (15/134)	5.9% (8/136)	5.2 (−1.5 to 12.4)

Abbreviations: ARC: augmented renal clearance; CI: confidence interval; EFU: early follow-up; ESBL: extended-spectrum β-lactamase; ICU: intensive care unit; HABP: hospital-acquired bacterial pneumonia; MITT: modified intent-to-treat; NA: not applicable; NRF: normal renal function; RI: renal impairment; and VABP: ventilator-associated bacterial pneumonia.

**Table 5:** Clinical response for cUTI, cIAI, and HABP/VABP populations.

Publication	Outcome	I/R % (n/N)	Colistin + Imipenem % (n/N)	Difference % (95 CI)
**RESTORE-IMI 1**				
**cUTI, cIAI and HABP/VABP (mMITT)**				
**Motsch (2020)** [28]	Favorable clinical response on-therapy on day 3	81.0% (17/21)	40.0% (4/10)	33.9 (7.4 to 61.1)
	Favorable clinical response at EOT	90.5% (19/21)	60.0% (6/10)	25.4 (3.1 to 53.6)
	Favorable clinical response at EFU	81.0% (17/21)	50.0% (5/10)	24.7 (3.8 to 51.4)
	Favorable clinical response on day 28	71.4% (15/21)	40.0% (4/10)	26.3 (1.3 to 51.5)
**cUTI, cIAI and HABP/VABP (SmMITT)**				
**Kaye (2020)** [30]	Favorable clinical response (day 28)	75.0% (21/28)	53.8% (7/13)	17.6 (−5.9 to 42.5)

Abbreviations: CI: confidence interval; cIAI: complicated intra-abdominal infection; cUTI: complicated urinary tract infection; EFU: early follow-up; EOT: end of treatment; HABP: hospital-acquired bacterial pneumonia; mMITT: microbiological modified intent-to-treat; SmMITT: supplemental microbiological modified intent-to-treat; and VABP: ventilator-associated bacterial pneumonia.

**Table 6:** Clinical response for cUTI and cIAI population.

Publication	Efficacy	I/R 250 mg % (n/N)	I/R 125 mg % (n/N)	Imipenem/Cilastatin + Placebo % (n/N)	Difference (95% CI)
**NCT01505634**					
**cUTI (ME)**					
**Sims (2017)** [32]	Favorable clinical response At EFU, 5–9 days after completion of all study therapy	89.10% (NA/70)	91.8% (NA/78)	93.4% (NA/80)	Relebactam 250 mg vs. placebo: −4.4 (−15.2 to 5.3) Relebactam 125 mg vs. placebo: −1.6 (−11.2 to 7.5)
	Favorable clinical response At LFU, 28–42 days after completion of all study therapy	88.70% (NA/70)	87.3% (NA/78)	88.2% (NA/79)	Relebactam 250 mg vs. placebo: −0.6 (−11.2 to 11.6) Relebactam 125 mg vs. placebo: −0.8 (−12.1 to 10.2)
**NCT01506271**					
**cIAI (ME)**					
**Lucasti (2016)** [33]	Favorable clinical response by Gram-negative aerobic bacilli at DCIV	97.3% (73/75)	100% (73/73)	94.4% (68/72)	Relebactam 250 mg vs. placebo: 2.9 (−4.4 to 11.2) Relebactam 125 mg vs. placebo: 5.6 (0.4 to 13.5
	Favorable clinical response by Gram-negative anaerobic bacilli at DCIV	91.7% (22/24)	100% (30/30)	96.3% (26/27)	Relebactam 250 mg vs. placebo: −4.6 (−23.0 to 11.4) Relebactam 125 mg vs. placebo: 3.7 (−8.1 to 18.5)
	Favorable clinical response Gram-positive aerobic cocci by at DCIV	100% (32/32)	97% (32/33)	97.1% (33/34)	Relebactam 250 mg vs. placebo: 2.9 (−8.1 to 15.1) Relebactam 125 mg vs. placebo: −0.1 (−12.9 to 12.4)

Abbreviations: CI: confidence interval; DCIV: discontinuation of IV therapy; EFU: early follow-up; HABP: hospital-acquired bacterial pneumonia; LFU: late follow-up; ME: microbiologically evaluable; mMITT: microbial modified intent-to-treat; NA: not applicable; and VABP: ventilator-associated bacterial pneumonia.

**Table 7:** Clinical response within HABP/VABP populations.

Publication	Outcome	I/R % (n/N)	Piperacillin + Tazobactam % (n/N)	Difference % (95 CI)
**RESTORE-IMI 2**				
**HABP/VABP (MITT)**				
**Titov (2021)** [34]	Favorable clinical response at EFU	61.0% (161/264)	55.8% (149/267)	5.0 (−3.2 to 13.2)
**HABP/VABP (subgroups by renal function)**				
**Roberts (2023)** [35]	Favorable clinical response at EFU among patients with NRF	66.0% (NA/103)	61.2% (NA/85)	4.8 (−8.9 to 18.6)
	Favorable clinical response at EFU among patients with ARC, ≥250	91.7% (NA/12)	44.4% (NA/9)	47.2 (7.6 to 76.2)
**HABP/VABP (subgroups by ICU admitted patients)**				
**Chen (2020)** [36]	Favorable clinical response at EFU for patients in ICU	58.9% (103/175)	54.5% (96/176)	4.3 (−6.1 to 14.6)
**HABP/VABP (MITT)**				
**EUCTR Trial Registry (2018)** [40]	Favorable clinical response at on-therapy visit 1 (day 3)	68.0% (NA/250)	64.7% (NA/252)	3.5 (−4.6 to 11.6)
	Favorable clinical response at on-therapy visit 2 (day 6)	83.5% (NA/236)	83.1% (NA/225)	0.5 (−6.3 to 7.4)
	Favorable clinical response at on-therapy visit 3 (day 10)	83.5% (NA/109)	80.4% (NA/102)	3.4 (−7.1 to 14.2)
	Favorable clinical response at EOT visit	74.2% (NA/264)	69.7% (NA/267)	4.4 (−3.1 to 12.0)
	Favorable clinical response on day 28	51.9% (NA/264)	50.6% (NA/267)	1.1 (−7.2 to 9.4)
**HABP/VABP (subgroups by pathogens)**				
**Losada (2020)** [38]	Clinical response at EFU among patients with ESBL positive	64.4% (NA/45)	60.0% (NA/35)	NA
**NCT03583333**				
**HABP/VABP (MITT)**				
**Li (2023)** [42]	Favorable clinical response at EOT	71.6% (96/134)	68.4% (93/136)	3.4 (−7.6 to 14.3)
	Favorable clinical response at EFU	50.7% (68/134)	47.8% (65/136)	3.1 (−8.7 to 14.9)

Abbreviations: ARC: augmented renal clearance; CI: confidence interval; EFU: early follow-up; EOT: end of treatment; ESBL: extended-spectrum beta-lactamase; HABP: hospital-acquired bacterial pneumonia; ICU: intensive care unit; MITT: modified intent-to-treat; NA: not applicable; NRF: normal renal function; and VABP: ventilator-associated bacterial pneumonia.

**Table 8:** Favorable microbiological responses in cUTI and cIAI populations.

Publication	Efficacy	I/R 250 mg % (n/N)	I/R 125 mg % (n/N)	Imipenem/Cilastatin + Placebo % (n/N)	Difference (95% CI)
**NCT01505634**					
**cUTI (ME)**					
**Sims (2017)** [32]	Favorable microbiological response At EFU, 5–9 days after completion of all study therapy	61.5% (NA/70)	68.1% (NA/78)	70.4% (NA/80)	Relebactam 250 mg vs. placebo: −8.9 (−24.6 to 7.1) Relebactam 125 mg vs. placebo: −2.4 (−17.4 to 12.8)
	Favorable microbiological response At LFU, 28–42 days after completion of all study therapy	68.30% (NA/70)	65.2% (NA/78)	62.5% (NA/79)	Relebactam 250 mg vs. placebo: 5.8 (−10.4 to 21.5) Relebactam 125 mg vs. placebo: 2.7 (−13.1 to 18.4)
**NCT01506271**					
**cIAI (ME)**					
**Lucasti (2016)** [33]	Favorable microbiological response At EFU (5 to 9 days after completion of IV study therapy)	97.4% (76/78)	97.6% (80/82)	97.5% (78/80)	Relebactam 250 mg vs. placebo: −0.1 (−6.7 to 6.4) Relebactam 125 mg vs. placebo: 0.1 (−6.3 to 6.5)
	Favorable microbiological response At LFU (28 to 42 days after completion of IV study therapy)	96.2% (75/78)	97.6% (80/82)	96.2% (75/78)	Relebactam 250 mg vs. placebo: 0.0 (−7.4 to 7.4) Relebactam 125 mg vs. placebo: 1.4 (−5.1 to 8.6)

Abbreviations: cIAI: complicated intra-abdominal infection; CI: confidence interval; EFU: early follow-up; IV: intravenous; LFU: late follow-up; ME: microbiologically evaluable; mg: milligram; and vs: versus.

**Table 9:** Favorable microbiological responses in HABP/VABP populations.

Publication	Outcome	I/R % (n/N)	Piperacillin + Tazobactam % (n/N)	Difference % (95 CI)
**RESTORE-IMI 2**				
**HABP/VABP (MITT)**				
**Titov (2021)** [34]	Favorable microbiologic response at EFU	67.9% (146/215)	61.9% (135/218)	6.2 (−2.7 to 15.0)
**HABP/VABP (subgroups by renal function)**				
**Roberts (2023)** [35]	Favorable microbiologic clinical response at EOT among patients with severe RI	57.1% (4/7)	42.9% (3/7)	14.3 (−36.4 to 58.4)
	Favorable microbiologic clinical response at EOT among patients with moderate RI	56.0% (28/50)	64.9% (24/37)	−8.9 (−28.6 to 12.1)
	Favorable microbiologic clinical response at EOT among patients with mild RI	80.4% (37/46)	74.1% (43/58)	6.3 (−10.5 to 22.1)
	Favorable microbiologic clinical response at EOT among patients with NRF	85.9% (67/78)	72.4% (55/76)	13.5 (0.7 to 26.4)
**HABP/VABP (MITT)**				
**EUCTR Trial Registry (2018)** [40]	Favorable microbiological response at EOT	87.1% (NA/140)	85.5% (NA/133)	2.5 (−5.5 to 11.0)
	Favorable microbiological response at EFU	89.9% (NA/121)	86.4% (NA/117)	4.7 (−4.0 to 14.1)
**HABP/VABP (subgroups by pathogens)**				
**Losada (2020)** [38]	Microbiologic response at EOT among patients with ESBL positive	82.2% (NA/45)	68.6% (NA/35)	NA
**NCT03583333**				
**HABP/VABP (MITT)**				
**Li (2023)** [42]	Favorable microbiological response at EOT	57.5% (46/80)	60.3% (44/73)	−2.4 (−17.8 to 13.1)
**HABP/VABP (ME)**				
**Li (2023)** [42]	Favorable microbiological response at EFU	80.0% (36/45)	78.4% (29/37)	1.4 (−16.5 to 19.8)
	Favorable microbiological response at EOT	71.9% (41/57)	74.5% (35/47)	−3.1 (−19.8 to 14.4)

Abbreviations: CI: confidence interval; EFU: early follow-up; EOT: end of treatment; HABP: hospital-acquired bacterial pneumonia; MITT: modified intent-to-treat; NA: not applicable; and VABP: ventilator-associated bacterial pneumonia.

## Data Availability

The full datasets generated and analyzed to inform the conclusions drawn within this manuscript during the current study are available from the corresponding author upon reasonable request.

## Supplementary Materials

The following supporting information can be downloaded at https://www.mdpi.com/article/10.3390/antibiotics15020170/s1, Reference [45] is cited in the Supplementary Materials.
