# Supplementary material for: Exploring the Effectiveness of Imipenem/Relebactam in Patients with Antimicrobial-Resistant Hospital-Acquired Infections: Findings from Systematic Literature Reviews
Source: Antibiotics (Basel). 2026 Feb 5;15(2):170. doi: 10.3390/antibiotics15020170 (PMC12937441; doi:10.3390/antibiotics15020170)
Supplement: Supplementary file 1 [file antibiotics-15-00170-s001.zip › antibiotics-4029864-supplementary.pdf]

**Study contents:**

| | |
|---|---|
| Title | Exploring the effectiveness of imipenem/relebactam in patients with cUTI, cIAI and HABP/VABP: findings from two systematic literature reviews |
| Authors | Ryan K. Shields, Ignacio Martin-Loeches, Emre Yucel, Shalini Bagga, Vaneet Pal Kaur Khurana, Prashant Soni, Prateek Das, Carolyn Cameron |
| Target Journal | Antibiotics (MDPI) |
| Communication Objectives | To evaluate the evidence for I/R versus alternative treatments from randomized controlled trials for HABP/VABP, cUTI (and pyelonephritis), and cIAI indications. |
| Manuscript Version | Version 5_0 |



Confidential - Not for Public Consumption or Distribution

## Table S1: Research question interpreted using PICOTS components

| | Requirements |
|---|---|
| **P - Population** | • Patients aged ≥ 18<br>• Require hospitalization and treatment for a bacterial infection for HABP/VABP, cUTI, cIAI |
| **I - Interventions** | • Imipenem/cilastatin + relebactam |
| **C - Comparators*** | • Placebo<br>• Any antibiotic, unrestricted selection of comparators |
| **O - Outcomes**** | • All-cause mortality<br>• Clinical/microbiological response on-therapy visits, (improved, persistence, progression, indeterminate)<br>• Clinical/microbiological response at end of therapy visits, (cure, improved, failure, indeterminate)<br>• Clinical/microbiological response at early follow-up and post-randomization (sustained cure, cure, failure, relapse, indeterminate)<br>• Serum creatinine level<br>• Creatinine clearance levels<br>• Hepatic function profile (total bilirubin, liver enzymes and total protein tests)<br>• Any adverse events<br>• Serious adverse events<br>• Treatment-related adverse events (specifically nephrotoxicity, hepatotoxicity and ototoxicity) |
| **T - Time** | • Inception to July 15, 2024 |
| **S - Study design** | • Phase 2/3 RCTs and controlled clinical trials (CCTs) (including blinded and open label studies) |
| **Language** | • English |

*Abbreviations: CCT: Controlled clinical trial; cIAI: Complicated Intra-Abdominal Infection; cUTI: Complicated urinary tract infection; HABP: Hospital-Acquired Bacterial Pneumonia; RCT: Randomized controlled trial; VABP: Ventilator-Associated Bacterial Pneumonia.*
** Studies investigating individual antibiotics as well as antibiotic combinations were of interest and were retained for full-text review*
***Data for these outcomes were extracted regardless of the time point at which they were collected; Searching time: From inception to July 15, 2024.*

## Table S2: List of data sources

| Data source | Source |
|---|---|
| **Databases** | • PubMed and MEDLINE (R) In process<br>• EMBASE (1989 – current)<br>• Cochrane Library (CENTRAL and CDSR) |
| **Conferences (2022 to 2024)** | • ESCMID Global<br>• ID Week<br>• ASM Microbe |



Confidential - Not for Public Consumption or Distribution

| Data source | Source |
|---|---|
| **Gray literature (Others)** | • Bibliographic searching of relevant SLRs |
| **Clinical trial searching** | • ClinicalTrials.gov website |

*Abbreviations: ASM: American Society for Microbiology; CDSR: Cochrane Database of Systematic Reviews; ESCMID: European Society of Clinical Microbiology and Infectious Diseases; ID: Infectious Disease; SLR: Systematic literature review.*



Confidential - Not for Public Consumption or Distribution

## Table S3: Search strategy for PubMed (2019 to July 15, 2024) for cUTI

| No | Search strings | Results |
|---|---|---|
| 1 | exp Urinary Tract Infections/ | 51,816 |
| 2 | exp Pyelonephritis/ | 15,170 |
| 3 | Catheter-Related Infections/ | 6,469 |
| 4 | (urinary tract infection$ or uti or utis).tw. | 44,893 |
| 5 | (pyelonephr$ or bacteriuria$).tw. | 18,365 |
| 6 | (catheter$ adj2 infection$).tw. | 4,594 |
| 7 | ((genitourin$ or ureter$ or ureth$ or urin$ or urolog$ or urogen$ or catheter) adj5 (infect$ or bacteria$ or microbiol$ or abscess$)).tw. | 69,364 |
| 8 | (urin$ adj5 (abnormal$ or obstruct$ or fistula$ or calcul$ or stricture$)).tw. | 19,651 |
| 9 | or/1-8 | 124,955 |
| 10 | (amikacin$ or amoxicillin$ or ampicillin$ or aztreonam$ or cefepime$ or cefiderocol$ or S-649266 or GSK-2696266 or cefoperazone$ or cefotaxime$ or ceftazidime$ or ceftibuten$ or ceftolozane$ or ceftriaxone$ or ciprofloxacin$ or colistin$ or colistimethate sodium or doripenem$ or eravacycline$ or TP-434 or ertapenem$ or faropenem$ or fosfomycin$ or gentamicin$ or imipenem$ or levofloxacin$ or meropenem$ or minocycline$ or netilmicin$ or piperacillin$ or plazomicin$ or ACHN- 490 or polymixin$ or prulifloxacin$ or temocillin$ or ticarcillin$ or tobramycin$ or trimethoprim$ or Relebactam$ or MK-7655).tw. | 148,312 |
| 11 | Randomized Controlled Trials as Topic/ | 170,834 |
| 12 | randomized controlled trial/ | 614,877 |
| 13 | Random Allocation/ | 107,324 |
| 14 | Double Blind Method/ | 178,967 |
| 15 | Single Blind Method/ | 33,594 |
| 16 | clinical trial/ | 539,098 |
| 17 | clinical trial, phase ii.pt. | 41,442 |
| 18 | clinical trial, phase iii.pt. | 22,829 |
| 19 | clinical trial, phase iv.pt. | 2,510 |
| 20 | controlled clinical trial.pt. | 95,530 |
| 21 | randomized controlled trial.pt. | 614,877 |
| 22 | multicenter study.pt. | 348,383 |
| 23 | clinical trial.pt. | 539,098 |
| 24 | exp Clinical Trials as topic/ | 393,271 |
| 25 | (clinical adj trial$).tw. | 434,584 |
| 26 | ((singl$ or doubl$ or treb$ or tripl$) adj (blind$3 or mask$3)).tw. | 185,301 |
| 27 | PLACEBOS/ | 35,965 |
| 28 | placebo$.tw. | 232,852 |
| 29 | randomly allocated.tw. | 32,183 |
| 30 | (allocated adj2 random$).tw. | 35,674 |
| 31 | or/11-30 | 1,873,206 |
| 32 | case report.tw. | 303,696 |
| 33 | letter/ | 1,207,660 |
| 34 | historical article/ | 370,291 |
| 35 | exp animals/ | 27,262,216 |



Confidential - Not for Public Consumption or Distribution

| No | Search strings | Results |
|---|---|---|
| 36 | humans/ | 22,031,262 |
| 37 | 35 and 36 | 22,031,262 |
| 38 | 35 not 37 | 5,230,954 |
| 39 | or/32-34,38 | 7,039,815 |
| 40 | 31 not 39 | 1,728,533 |
| 41 | (2019$ or 2020$ or 2021$ or 2022$ or 2023$ or 2024$).ez,ep,dt. | 5,268,861 |
| 42 | ("2019" or "2020" or "2021" or "2022" or "2023" or "2024").yr. | 5,437,387 |
| 43 | 9 and 10 and 40 | 2,163 |
| 44 | 43 and (41 or 42) | 255 |
| 45 | limit 44 to english language | 238 |



Confidential - Not for Public Consumption or Distribution

## Table S4: Search strategy for MEDLINE (2019 to July 15, 2024) for cIAI

| No | Search strings | Results |
|---|---|---|
| 1 | exp Intraabdominal Infections/ | 50,925 |
| 2 | exp abdominal abscess/ | 12,977 |
| 3 | (intra abdominal infection$ or intra-abdominal infection$ or intraabdominal infection$).tw. | 3,293 |
| 4 | ((intraabdominal or intra-abdominal or abdominal) adj2 (infection$ or abscess$)).tw. | 9,868 |
| 5 | appendicitis.tw. | 23,008 |
| 6 | diverticulitis.tw. | 7,711 |
| 7 | peritonitis.tw. | 35,132 |
| 8 | or/1-7 | 96,107 |
| 9 | amikacin$.tw. | 11,894 |
| 10 | amoxicillin$.tw. | 19,891 |
| 11 | ampicillin$.tw. | 27,903 |
| 12 | aztreonam$.tw. | 3,858 |
| 13 | cefepime$.tw. | 4,615 |
| 14 | cefoperazone$.tw. | 3,003 |
| 15 | cefotaxime$.tw. | 9,917 |
| 16 | ceftazidime$.tw. | 11,912 |
| 17 | ceftolozane$.tw. | 881 |
| 18 | ceftriaxone$.tw. | 14,090 |
| 19 | cefuroxime$.tw. | 5,051 |
| 20 | ciprofloxacin$.tw. | 33,295 |
| 21 | colistin$.tw. | 9,963 |
| 22 | colistimethate sodium$.tw. | 186 |
| 23 | doripenem$.tw. | 794 |
| 24 | (Eravacycline$ or TP-434).tw. | 281 |
| 25 | ertapenem$.tw. | 2,167 |
| 26 | gentamicin$.tw. | 28,684 |
| 27 | imipenem$.tw. | 13,100 |
| 28 | levofloxacin$.tw. | 10,502 |
| 29 | meropenem$.tw. | 10,511 |
| 30 | moxifloxacin$.tw. | 6,054 |
| 31 | piperacillin$.tw. | 9,201 |
| 32 | polymixin$.tw. | 388 |
| 33 | ticarcillin$.tw. | 2,067 |
| 34 | tigecycline$.tw. | 4,862 |



Confidential - Not for Public Consumption or Distribution

| No | Search strings | Results |
|---|---|---|
| 35 | tobramycin$.tw. | 7,947 |
| 36 | or/9-35 | 157,458 |
| 37 | Randomized Controlled Trials as Topic/ | 170,779 |
| 38 | randomized controlled trial/ | 615,041 |
| 39 | Random Allocation/ | 107,314 |
| 40 | Double Blind Method/ | 178,943 |
| 41 | Single Blind Method/ | 33,589 |
| 42 | clinical trial/ | 540,025 |
| 43 | clinical trial, phase ii.pt. | 41,429 |
| 44 | clinical trial, phase iii.pt. | 22,826 |
| 45 | clinical trial, phase iv.pt. | 2,510 |
| 46 | controlled clinical trial.pt. | 95,551 |
| 47 | randomized controlled trial.pt. | 615,041 |
| 48 | multicenter study.pt. | 348,268 |
| 49 | clinical trial.pt. | 540,025 |
| 50 | exp Clinical Trials as topic/ | 393,195 |
| 51 | (clinical adj trial$).tw. | 514,768 |
| 52 | ((singl$ or doubl$ or treb$ or tripl$) adj (blind$3 or mask$3)).tw. | 205,720 |
| 53 | PLACEBOS/ | 35,965 |
| 54 | placebo$.tw. | 257,059 |
| 55 | randomly allocated.tw. | 38,804 |
| 56 | (allocated adj2 random$).tw. | 42,734 |
| 57 | or/37-56 | 1,982,730 |
| 58 | case report.tw. | 428,684 |
| 59 | letter/ | 1,257,738 |
| 60 | historical article/ | 370,357 |
| 61 | exp animals/ | 27,258,019 |
| 62 | humans/ | 22,027,633 |
| 63 | 61 and 62 | 22,027,633 |
| 64 | 61 not 63 | 5,230,386 |
| 65 | or/58-60,64 | 7,213,662 |
| 66 | 57 not 65 | 1,836,933 |
| 67 | 8 and 36 and 66 | 927 |
| 68 | limit 67 to english language | 831 |
| 69 | (2017$ or 2018$ or 2019$ or 2020$ or 2021$ or 2022$ or 2023$ or 2024$).ez,ep,dt. | 10,643,358 |
| 70 | ("2017" or "2018" or "2019" or "2020" or "2021" or "2022" or "2023" or "2024").yr. | 10,454,463 |



Confidential - Not for Public Consumption or Distribution

| No | Search strings | Results |
|---|---|---|
| 71 | 68 and (69 or 70) | 148 |



Confidential - Not for Public Consumption or Distribution

**Table S5: Search strategy for EMBASE (2019 to July 15, 2024) for cUTI**

| No | Search strings | Results |
|---|---|---|
| 1 | exp urinary tract infection/ | 147,174 |
| 2 | exp pyelonephritis/ | 25,590 |
| 3 | catheter infection/ or catheter associated urinary tract infection/ | 22,563 |
| 4 | (urinary tract infection$ or uti or utis).tw. | 84,681 |
| 5 | (pyelonephr$ or bacteriuria$).tw. | 24,570 |
| 6 | (catheter$ adj2 infection$).tw. | 7,880 |
| 7 | ((genitourin$ or ureter$ or ureth$ or urin$ or urolog$ or urogen$ or catheter) adj5 (infect$ or bacteria$ or microbiol$ or abscess$)).tw. | 119,542 |
| 8 | (urin$ adj5 (abnormal$ or obstruct$ or fistula$ or calcul$ or stricture$)).tw. | 31,008 |
| 9 | or/1-8 | 253,049 |
| 10 | (amikacin$ or amoxicillin$ or ampicillin$ or aztreonam$ or cefepime$ or cefiderocol$ or S-649266 or GSK-2696266 or cefoperazone$ or cefotaxime$ or ceftazidime$ or ceftibuten$ or ceftolozane$ or ceftriaxone$ or ciprofloxacin$ or colistin$ or colistimethate sodium or doripenem$ or eravacycline$ or TP-434 or ertapenem$ or faropenem$ or fosfomycin$ or gentamicin$ or imipenem$ or levofloxacin$ or meropenem$ or minocycline$ or netilmicin$ or piperacillin$ or plazomicin$ or ACHN- 490 or polymixin$ or prulifloxacin$ or temocillin$ or ticarcillin$ or tobramycin$ or trimethoprim$ or Relebactam$ or MK-7655).tw. | 240,053 |
| 11 | clinical Trial/ | 1,083,397 |
| 12 | randomized controlled trial/ | 824,777 |
| 13 | Randomization/ | 99,411 |
| 14 | single blind procedure/ | 54,989 |
| 15 | double blind procedure/ | 219,717 |
| 16 | crossover procedure/ | 78,244 |
| 17 | placebo/ | 413,583 |
| 18 | Randomi?ed controlled trial$.tw. | 347,683 |
| 19 | rct.tw. | 57,936 |
| 20 | Random allocation.tw. | 2,711 |
| 21 | Randomly allocated.tw. | 47,841 |
| 22 | Allocated randomly.tw. | 3,060 |
| 23 | (allocated adj2 random).tw. | 967 |
| 24 | Single blind$.tw. | 33,235 |
| 25 | Double blind$.tw. | 252,550 |
| 26 | ((treble or triple) adj blind$).tw. | 2,126 |
| 27 | placebo$.tw. | 380,196 |



Confidential - Not for Public Consumption or Distribution

| No | Search strings | Results |
|---|---|---|
| 28 | Prospective study/ | 921,591 |
| 29 | or/11-28 | 2,877,996 |
| 30 | case study/ | 101,240 |
| 31 | case report.tw. | 571,983 |
| 32 | abstract report/ or letter/ | 1,331,429 |
| 33 | (rat or rats or mouse or mice or swine or porcine or murine or sheep or lambs or pigs or piglets or rabbit or rabbits or cat or cats or dog or dogs or cattle or bovine or monkey or monkeys or trout or marmoset$).ti. and animal experiment/ | 1,254,535 |
| 34 | 'animal experiment'/ not ('human experiment'/ or 'human'/) | 2,638,045 |
| 35 | or/30-34 | 4,652,595 |
| 36 | (2019$ or 2020$ or 2021$ or 2022$ or 2023$ or 2024$).em. | 13,126,305 |
| 37 | ("2019" or "2020" or "2021" or "2022" or "2023" or "2024").yr. | 9,900,121 |
| 38 | 29 not 35 | 2,766,262 |
| 39 | 9 and 10 and 38 | 3,403 |
| 40 | 39 and (36 or 37) | 1,095 |
| 41 | limit 40 to english language | 1,060 |



Confidential - Not for Public Consumption or Distribution

## Table S6: Search strategy for EMBASE (2019 to July 15, 2024) for cIAI

| No | Search strings | Results |
|---|---|---|
| 1 | exp abdominal Infection/ | 34,283 |
| 2 | exp appendicitis/ | 25,810 |
| 3 | exp diverticulitis/ | 9,656 |
| 4 | exp peritonitis/ | 52,986 |
| 5 | (intra abdominal infection$ or intra-abdominal infection$ or intraabdominal infection$).tw. | 4,233 |
| 6 | ((intraabdominal or intra-abdominal or abdominal) adj2 (infection$ or abscess$)).tw. | 12,630 |
| 7 | appendicitis.tw. | 21,384 |
| 8 | diverticulitis.tw. | 9,609 |
| 9 | peritonitis.tw. | 35,533 |
| 10 | or/1-9 | 118,172 |
| 11 | amikacin$.tw. | 14,412 |
| 12 | amoxicillin$.tw. | 27,892 |
| 13 | ampicillin$.tw. | 25,373 |
| 14 | aztreonam$.tw. | 4,161 |
| 15 | cefepime$.tw. | 7,942 |
| 16 | cefoperazone$.tw. | 2,652 |
| 17 | cefotaxime$.tw. | 9,644 |
| 18 | ceftazidime$.tw. | 13,919 |
| 19 | ceftolozane$.tw. | 1,306 |
| 20 | ceftriaxone$.tw. | 21,118 |
| 21 | cefuroxime$.tw. | 5,332 |
| 22 | ciprofloxacin$.tw. | 40,882 |
| 23 | colistin$.tw. | 12,209 |
| 24 | colistimethate sodium$.tw. | 313 |
| 25 | doripenem$.tw. | 1,209 |
| 26 | (Eravacycline$ or TP-434).tw. | 414 |
| 27 | ertapenem$.tw. | 3,567 |
| 28 | gentamicin$.tw. | 26,616 |
| 29 | imipenem$.tw. | 16,513 |
| 30 | levofloxacin$.tw. | 16,948 |
| 31 | meropenem$.tw. | 17,099 |
| 32 | moxifloxacin$.tw. | 8,823 |
| 33 | piperacillin$.tw. | 13,629 |
| 34 | polymixin$.tw. | 386 |
| 35 | ticarcillin$.tw. | 1,257 |
| 36 | tigecycline$.tw. | 6,926 |
| 37 | tobramycin$.tw. | 7,423 |
| 38 | or/11-37 | 180,583 |
| 39 | clinical Trial/ | 946,761 |
| 40 | randomized controlled trial/ | 777,526 |



Confidential - Not for Public Consumption or Distribution

| No | Search strings | Results |
|---|---|---|
| 41 | Randomization/ | 91,084 |
| 42 | single blind procedure/ | 53,458 |
| 43 | double blind procedure/ | 193,038 |
| 44 | crossover procedure/ | 73,715 |
| 45 | placebo/ | 360,453 |
| 46 | Randomi?ed controlled trial$.tw. | 343,543 |
| 47 | rct.tw. | 57,397 |
| 48 | Random allocation.tw. | 2,383 |
| 49 | Randomly allocated.tw. | 43,669 |
| 50 | Allocated randomly.tw. | 2,403 |
| 51 | (allocated adj2 random).tw. | 463 |
| 52 | Single blind$.tw. | 29,715 |
| 53 | Double blind$.tw. | 206,529 |
| 54 | ((treble or triple) adj blind$).tw. | 2,046 |
| 55 | placebo$.tw. | 330,388 |
| 56 | Prospective study/ | 900,507 |
| 57 | or/39-56 | 2,646,205 |
| 58 | case study/ | 98,571 |
| 59 | case report.tw. | 505,811 |
| 60 | abstract report/ or letter/ | 973,249 |
| 61 | (rat or rats or mouse or mice or swine or porcine or murine or sheep or lambs or pigs or piglets or rabbit or rabbits or cat or cats or dog or dogs or cattle or bovine or monkey or monkeys or trout or marmoset$).ti. and animal experiment/ | 876,810 |
| 62 | 'animal experiment'/ not ('human experiment'/ or 'human'/) | 1,825,511 |
| 63 | or/58-62 | 3,446,696 |
| 64 | 10 and 38 and 57 | 1,105 |
| 65 | 64 not 63 | 1,076 |
| 66 | (2017$ or 2018$ or 2019$ or 2020$ or 2021$ or 2022$ or 2023$ or 2024$).em. | 15,784,905 |
| 67 | ("2017" or "2018" or "2019" or "2020" or "2021" or "2022" or "2023" or "2024").yr. | 12,968,884 |
| 68 | 65 and (66 or 67) | 454 |
| 69 | limit 68 to english language | 445 |



Confidential - Not for Public Consumption or Distribution

**Table S7: Search strategy for Cochrane Database of Systematic Reviews (2019 to July 15, 2024) for cUTI**

| No | Search strings | Results |
|---|---|---|
| #1 | MeSH descriptor: [Urinary Tract Infections] explode all trees | 3,305 |
| #2 | MeSH descriptor: [Pyelonephritis] explode all trees | 313 |
| #3 | MeSH descriptor: [Catheter-Related Infections] explode all trees | 550 |
| #4 | (("urinary tract" NEXT infection*) OR uti OR utis):ti,ab,kw | 10,831 |
| #5 | (pyelonephr* OR bacteriuria*):ti,ab,kw | 2,270 |
| #6 | (catheter* NEAR/2 infection*):ti,ab,kw | 1,873 |
| #7 | ((genitourin* OR ureter* OR ureth* OR urin* OR urolog* OR urogen* OR catheter) NEAR/5 (infect* OR bacteria* OR microbiol* OR abscess*)):ti,ab,kw | 14,348 |
| #8 | (urin* NEAR/5 (abnormal* OR obstruct* OR fistula* OR calcul* OR stricture*)):ti,ab,kw | 2,559 |
| #9 | 37-#8 | 17,738 |
| #10 | (amikacin* OR amoxicillin* OR ampicillin* OR aztreonam* OR cefepime* OR cefiderocol* OR "S 649266" OR "GSK 2696266" OR cefoperazone* OR cefotaxime* OR ceftazidime* OR ceftibuten* OR ceftolozane* OR ceftriaxone* OR ciprofloxacin* OR colistin* OR "colistimethate sodium" OR doripenem* OR eravacycline* OR "TP 434" OR ertapenem* OR faropenem* OR fosfomycin* OR gentamicin* OR imipenem* OR levofloxacin* OR meropenem* OR minocycline* OR netilmicin* OR piperacillin* OR plazomicin* OR "ACHN 490" OR polymixin* OR prulifloxacin* OR temocillin* OR ticarcillin* OR tobramycin* OR trimethoprim* OR Relebactam* OR "MK 7655"):ti,ab,kw | 24,076 |
| #11 | #9 AND #10 with Cochrane Library publication date Between May 2019 and Jun 2024, in Cochrane Reviews | 4 |

**Table S8: Search strategy for Cochrane Database of Systematic Reviews (2019 to July 15, 2024) for cIAI**

| No | Search strings | Results |
|---|---|---|
| #1 | (("intra abdominal" NEXT infection*) OR (intraabdominal NEXT infection*)):ti,ab,kw | 608 |
| #2 | (abdominal NEAR/2 (infection* OR abscess*)):ti,ab,kw | 1722 |
| #3 | appendicitis:ti,ab,kw | 1976 |
| #4 | diverticulitis:ti,ab,kw | 599 |
| #5 | peritonitis:ti,ab,kw | 2580 |
| #6 | 37-#5 | 6179 |
| #7 | (amikacin* OR amoxicillin* OR ampicillin* OR aztreonam*):ti,ab,kw | 9798 |
| #8 | (cefepime* OR cefoperazone* OR cefotaxime* OR ceftazidime*):ti,ab,kw | 2644 |



Confidential - Not for Public Consumption or Distribution

| No | Search strings | Results |
|---|---|---|
| #9 | (ceftolozane* OR ceftriaxone* OR cefuroxime* OR ciprofloxacin*):ti,ab,kw | 5680 |
| #10 | (colistin* OR (colistimethate NEXT sodium*)):ti,ab,kw | 605 |
| #11 | (doripenem* OR eravacycline* OR "TP 434" OR ertapenem*):ti,ab,kw | 349 |
| #12 | (gentamicin* OR imipenem* OR levofloxacin* OR meropenem* OR moxifloxacin*):ti,ab,kw | 7315 |
| #13 | (piperacillin* OR polymixin* OR ticarcillin* OR tigecycline*):ti,ab,kw | 1475 |
| #14 | tobramycin*:ti,ab,kw | 1551 |
| #15 | 37-#14 | 23080 |
| #16 | #6 AND #15 with Cochrane Library publication date Between Apr 2017 and Jul 2024, in Cochrane Reviews | 3 |



Confidential - Not for Public Consumption or Distribution

## Table S9: Search strategy for Cochrane Central Register of Controlled Trials (CENTRAL) (2019 to July 15, 2024) for cUTI

| No | Search strings | Results |
|---|---|---|
| #1 | MeSH descriptor: [Urinary Tract Infections] explode all trees | 3,305 |
| #2 | MeSH descriptor: [Pyelonephritis] explode all trees | 313 |
| #3 | MeSH descriptor: [Catheter-Related Infections] explode all trees | 550 |
| #4 | (("urinary tract" NEXT infection*) OR uti OR utis):ti,ab,kw | 10,831 |
| #5 | (pyelonephr* OR bacteriuria*):ti,ab,kw | 2,270 |
| #6 | (catheter* NEAR/2 infection*):ti,ab,kw | 1,873 |
| #7 | ((genitourin* OR ureter* OR ureth* OR urin* OR urolog* OR urogen* OR catheter) NEAR/5 (infect* OR bacteria* OR microbiol* OR abscess*)):ti,ab,kw | 14,348 |
| #8 | (urin* NEAR/5 (abnormal* OR obstruct* OR fistula* OR calcul* OR stricture*)):ti,ab,kw | 2,559 |
| #9 | 37-#8 | 17,738 |
| #10 | (amikacin* OR amoxicillin* OR ampicillin* OR aztreonam* OR cefepime* OR cefiderocol* OR “S 649266” OR “GSK 2696266” OR cefoperazone* OR cefotaxime* OR ceftazidime* OR ceftibuten* OR ceftolozane* OR ceftriaxone* OR ciprofloxacin* OR colistin* OR “colistimethate sodium” OR doripenem* OR eravacycline* OR “TP 434” OR ertapenem* OR faropenem* OR fosfomycin* OR gentamicin* OR imipenem* OR levofloxacin* OR meropenem* OR minocycline* OR netilmicin* OR piperacillin* OR plazomicin* OR “ACHN 490” OR polymixin* OR prulifloxacin* OR temocillin* OR ticarcillin* OR tobramycin* OR trimethoprim* OR Relebactam* OR “MK 7655”):ti,ab,kw | 24,076 |
| #11 | #9 AND #10 with Publication Year from 2019 to 2024, in Trials | 449 |

## Table S10: Search strategy for Cochrane Central Register of Controlled Trials (CENTRAL) (2019 to July 15, 2024) for cIAI

| No | Search strings | Results |
|---|---|---|
| #1 | (("intra abdominal" NEXT infection*) OR (intraabdominal NEXT infection*)):ti,ab,kw | 608 |
| #2 | (abdominal NEAR/2 (infection* OR abscess*)):ti,ab,kw | 1722 |
| #3 | appendicitis:ti,ab,kw | 1976 |
| #4 | diverticulitis:ti,ab,kw | 599 |
| #5 | peritonitis:ti,ab,kw | 2580 |
| #6 | 37-#5 | 6179 |
| #7 | (amikacin* OR amoxicillin* OR ampicillin* OR aztreonam*):ti,ab,kw | 9798 |
| #8 | (cefepime* OR cefoperazone* OR cefotaxime* OR ceftazidime*):ti,ab,kw | 2644 |
| #9 | (ceftolozane* OR ceftriaxone* OR cefuroxime* OR ciprofloxacin*):ti,ab,kw | 5680 |
| #10 | (colistin* OR (colistimethate NEXT sodium*)):ti,ab,kw | 605 |
| #11 | (doripenem* OR eravacycline* OR "TP 434" OR ertapenem*):ti,ab,kw | 349 |



Confidential - Not for Public Consumption or Distribution

| No | Search strings | Results |
|---|---|---|
| #12 | (gentamicin* OR imipenem* OR levofloxacin* OR meropenem* OR moxifloxacin*):ti,ab,kw | 7315 |
| #13 | (piperacillin* OR polymixin* OR ticarcillin* OR tigecycline*):ti,ab,kw | 1475 |
| #14 | tobramycin*:ti,ab,kw | 1551 |
| #15 | 37-#14 | 23080 |
| #16 | #6 AND #15 with Publication Year from 2017 to 2024, in Trials | 231 |

**Table S11: Search strategy for MEDLINE (1946 to April 05, 2017) for cUTI**

| No | Search strings | Results |
|---|---|---|
| 1 | exp urinary tract infection/ | 42639 |
| 2 | exp Pyelonephritis/ | 14233 |
| 3 | exp Catheter-Related Infections/ | 3409 |
| 4 | (urinary tract infection$ or uti or utis).tw. | 34972 |
| 5 | pyelonephr$.tw. | 12082 |
| 6 | bacteriuria$.tw. | 5405 |
| 7 | (catheter$ adj2 infection$).tw. | 3878 |
| 8 | ((genitourin$ or ureter$ or ureth$ or urin$ or urolog$ or urogen$ or catheter) adj5 (infect$ or bacteria$ or microbiol$ or abcess$)).tw. | 55282 |
| 9 | (urin$ adj5 (abnormal$ or obstruct$ or fistula$ or calcul$ or stricture$)).tw. | 17462 |
| 10 | or/1-9 | 105320 |
| 11 | amikacin$.tw. | 8198 |
| 12 | amoxicillin$.tw. | 12669 |
| 13 | ampicillin$.tw. | 20739 |
| 14 | aztreonam$.tw. | 2767 |
| 15 | cefepime$.tw. | 2695 |
| 16 | (cefiderocol$ or S-649266 or GSK-2696266).tw. | 10 |
| 17 | cefoperazone$.tw. | 2399 |
| 18 | cefotaxime$.tw. | 7543 |
| 19 | ceftazidime$.tw. | 7796 |
| 20 | ceftibuten$.tw. | 288 |
| 21 | ceftolozane$.tw. | 154 |
| 22 | ceftriaxone$.tw. | 8926 |
| 23 | ciprofloxacin$.tw. | 21957 |
| 24 | colistin$.tw. | 3953 |
| 25 | colistimethate sodium.tw. | 97 |



Confidential - Not for Public Consumption or Distribution

| No | Search strings | Results |
|---|---|---|
| 26 | doripenem$.tw. | 542 |
| 27 | (eravacycline$ or TP-434).tw. | 40 |
| 28 | ertapenem$.tw. | 1147 |
| 29 | faropenem$.tw. | 160 |
| 30 | fosfomycin$.tw. | 2313 |
| 31 | gentamicin$.tw. | 22259 |
| 32 | imipenem$.tw. | 9069 |
| 33 | levofloxacin$.tw. | 6199 |
| 34 | meropenem$.tw. | 4766 |
| 35 | minocycline$.tw. | 5747 |
| 36 | netilmicin$.tw. | 1758 |
| 37 | piperacillin$.tw. | 5835 |
| 38 | (plazomicin$ or ACHN-490).tw. | 46 |
| 39 | polymixin$.tw. | 340 |
| 40 | prulifloxacin$.tw. | 135 |
| 41 | temocillin$.tw. | 236 |
| 42 | ticarcillin$.tw. | 1936 |
| 43 | tobramycin$.tw. | 6289 |
| 44 | trimethoprim$.tw. | 14787 |
| 45 | or/11-44 | 117979 |
| 46 | Randomized Controlled Trials as Topic/ | 111711 |
| 47 | randomized controlled trial/ | 457480 |
| 48 | Random Allocation/ | 91777 |
| 49 | Double Blind Method/ | 145956 |
| 50 | Single Blind Method/ | 24185 |
| 51 | clinical trial/ | 518663 |
| 52 | clinical trial, phase ii.pt. | 29780 |
| 53 | clinical trial, phase iii.pt. | 13503 |
| 54 | clinical trial, phase iv.pt. | 1443 |
| 55 | controlled clinical trial.pt. | 93386 |
| 56 | randomized controlled trial.pt. | 457480 |
| 57 | multicenter study.pt. | 223536 |
| 58 | clinical trial.pt. | 518663 |
| 59 | exp Clinical Trials as topic/ | 309936 |



Confidential - Not for Public Consumption or Distribution

| No | Search strings | Results |
|---|---|---|
| 60 | (clinical adj trial$).tw. | 294075 |
| 61 | ((singl$ or doubl$ or treb$ or tripl$) adj (blind$3 or mask$3)).tw. | 155976 |
| 62 | PLACEBOS/ | 34759 |
| 63 | placebo$.tw. | 193280 |
| 64 | randomly allocated.tw. | 22958 |
| 65 | (allocated adj2 random$).tw. | 25927 |
| 66 | or/46-65 | 1419984 |
| 67 | case report.tw. | 257176 |
| 68 | letter/ | 965438 |
| 69 | historical article/ | 344536 |
| 70 | animal/ | 6052916 |
| 71 | human/ | 16688414 |
| 72 | 70 and 71 | 1719309 |
| 73 | 70 not 72 | 4333607 |
| 74 | or/67-69,73 | 5842889 |
| 75 | 66 not 74 | 1307485 |
| 76 | 10 and 45 and 75 | 1889 |
| 77 | limit 76 to english language | 1543 |



Confidential - Not for Public Consumption or Distribution

## Table S12: Search strategy for EMBASE (1946 to April 05, 2017) for cUTI

| No | Search strings | Results |
|---|---|---|
| 1 | exp urinary tract infection/ | 76397 |
| 2 | exp pyelonephritis/ | 13561 |
| 3 | exp bacteriuria/ | 4356 |
| 4 | exp catheter infection/ | 14815 |
| 5 | (urinary tract infection$ or uti or utis).tw. | 42781 |
| 6 | pyelonephr$.tw. | 9287 |
| 7 | bacteriuria$.tw. | 4175 |
| 8 | (catheter$ adj2 infection$).tw. | 5194 |
| 9 | ((genitourin$ or ureter$ or ureth$ or urin$ or urolog$ or urogen$ or catheter) adj5 (infect$ or bacteria$ or microbiol$ or abcess$)).tw. | 62883 |
| 10 | (urin$ adj5 (abnormal$ or obstruct$ or fistula$ or calcul$ or stricture$)).tw. | 18131 |
| 11 | or/1-10 | 134215 |
| 12 | amikacin$.tw. | 9476 |
| 13 | amoxicillin$.tw. | 17017 |
| 14 | ampicillin$.tw. | 18541 |
| 15 | aztreonam$.tw. | 3246 |
| 16 | cefepime$.tw. | 4188 |
| 17 | (cefiderocol$ or S-649266 or GSK-2696266).tw. | 21 |
| 18 | cefoperazone$.tw. | 2211 |
| 19 | cefotaxime$.tw. | 7728 |
| 20 | ceftazidime$.tw. | 9388 |
| 21 | ceftibuten$.tw. | 388 |
| 22 | ceftolozane$.tw. | 170 |
| 23 | ceftriaxone$.tw. | 12115 |
| 24 | ciprofloxacin$.tw. | 28635 |
| 25 | colistin$.tw. | 4850 |
| 26 | colistimethate sodium.tw. | 174 |
| 27 | doripenem$.tw. | 867 |
| 28 | (eravacycline$ or TP-434).tw. | 80 |
| 29 | ertapenem$.tw. | 1851 |
| 30 | faropenem$.tw. | 243 |
| 31 | fosfomycin$.tw. | 2510 |



Confidential - Not for Public Consumption or Distribution

| No | Search strings | Results |
|---|---|---|
| 32 | gentamicin$.tw. | 20271 |
| 33 | imipenem$.tw. | 12181 |
| 34 | levofloxacin$.tw. | 9645 |
| 35 | meropenem$.tw. | 7859 |
| 36 | minocycline$.tw. | 6834 |
| 37 | netilmicin$.tw. | 1351 |
| 38 | piperacillin$.tw. | 7905 |
| 39 | (plazomicin$ or ACHN-490).tw. | 106 |
| 40 | polymixin$.tw. | 362 |
| 41 | prulifloxacin$.tw. | 210 |
| 42 | temocillin$.tw. | 191 |
| 43 | ticarcillin$.tw. | 1505 |
| 44 | tobramycin$.tw. | 5822 |
| 45 | trimethoprim$.tw. | 14287 |
| 46 | or/12-45 | 129577 |
| 47 | Clinical trial/ | 1004577 |
| 48 | Randomized controlled trial/ | 471107 |
| 49 | Randomization/ | 83111 |
| 50 | Single blind procedure/ | 30582 |
| 51 | Double blind procedure/ | 133120 |
| 52 | Crossover procedure/ | 55916 |
| 53 | Placebo/ | 290718 |
| 54 | Randomi?ed controlled trial$.tw. | 157276 |
| 55 | Rct.tw. | 23689 |
| 56 | Random allocation.tw. | 1539 |
| 57 | Randomly allocated.tw. | 26054 |
| 58 | Allocated randomly.tw. | 2096 |
| 59 | (allocated adj2 random).tw. | 580 |
| 60 | Single blind$.tw. | 18010 |
| 61 | Double blind$.tw. | 154953 |
| 62 | ((treble or triple) adj blind$).tw. | 652 |
| 63 | Placebo$.tw. | 233802 |
| 64 | Prospective study/ | 401829 |
| 65 | or/47-64 | 1774206 |



Confidential - Not for Public Consumption or Distribution

| No | Search strings | Results |
|---|---|---|
| 66 | Case study/ | 95104 |
| 67 | Case report.tw. | 294676 |
| 68 | Abstract report/ or letter/ | 838379 |
| 69 | animal/ | 975357 |
| 70 | human/ | 15790687 |
| 71 | 69 and 70 | 283233 |
| 72 | 69 not 71 | 692124 |
| 73 | or/66-68,72 | 1902628 |
| 74 | 65 not 73 | 1694815 |
| 75 | 11 and 46 and 74 | 2182 |
| 76 | limit 75 to english language | 1785 |



Confidential - Not for Public Consumption or Distribution

**Table S13: Search strategy for COCHRANE LIBRARY (CENTRAL: November 2016 and CDSR: 2005 to April 04, 2017) for cUTI**

| No | Search strings | Results |
|---|---|---|
| 1 | (urinary tract infection$ or uti or utis).tw. | 3842 |
| 2 | pyelonephr$.tw. | 407 |
| 3 | bacteriuria$.tw. | 731 |
| 4 | (catheter$ adj2 infection$).tw. | 632 |
| 5 | ((genitourin$ or ureter$ or ureth$ or urin$ or urolog$ or urogen$ or catheter) adj5 (infect$ or bacteria$ or microbiol$ or abcess$)).tw. | 5498 |
| 6 | (urin$ adj5 (abnormal$ or obstruct$ or fistula$ or calcul$ or stricture$)).tw. | 1109 |
| 7 | or/1-6 | 7016 |
| 8 | amikacin$.tw. | 669 |
| 9 | amoxicillin$.tw. | 3015 |
| 10 | ampicillin$.tw. | 1373 |
| 11 | aztreonam$.tw. | 288 |
| 12 | cefepime$.tw. | 208 |
| 13 | (cefiderocol$ or S-649266 or GSK-2696266).tw. | 2 |
| 14 | cefoperazone$.tw. | 233 |
| 15 | cefotaxime$.tw. | 667 |
| 16 | ceftazidime$.tw. | 794 |
| 17 | ceftibuten$.tw. | 88 |
| 18 | ceftolozane$.tw. | 19 |
| 19 | ceftriaxone$.tw. | 1072 |
| 20 | ciprofloxacin$.tw. | 1817 |
| 21 | colistin$.tw. | 209 |
| 22 | colistimethate sodium.tw. | 22 |
| 23 | doripenem$.tw. | 40 |
| 24 | (eravacycline$ or TP-434).tw. | 3 |
| 25 | ertapenem$.tw. | 85 |
| 26 | faropenem$.tw. | 17 |
| 27 | fosfomycin$.tw. | 134 |
| 28 | gentamicin$.tw. | 1474 |
| 29 | imipenem$.tw. | 463 |
| 30 | levofloxacin$.tw. | 871 |
| 31 | meropenem$.tw. | 294 |



Confidential - Not for Public Consumption or Distribution

| No | Search strings | Results |
|---|---|---|
| 32 | minocycline$.tw. | 585 |
| 33 | netilmicin$.tw. | 250 |
| 34 | piperacillin$.tw. | 620 |
| 35 | (plazomicin$ or ACHN-490).tw. | 2 |
| 36 | polymixin$.tw. | 19 |
| 37 | prulifloxacin$.tw. | 39 |
| 38 | temocillin$.tw. | 20 |
| 39 | ticarcillin$.tw. | 241 |
| 40 | tobramycin$.tw. | 1085 |
| 41 | trimethoprim$.tw. | 1258 |
| 42 | or/8-41 | 13135 |
| 43 | 7 and 42 | 1554 |
| 44 | limit 43 to english language [Limit not valid in CDSR; records were retained] | 1105 |

## Table S14: Search strategy for MEDLINE and EMBASE (1946 to May 17, 2019) for cUTI

| No | Search strings | Results |
|---|---|---|
| 1 | exp urinary tract infection/ or exp Pyelonephritis/ or exp Catheter-Related Infections/ or (urinary tract infection$ or uti or utis).tw. or pyelonephr$.tw. or bacteriuria$.tw. or (catheter$ adj2 infection$).tw. or ((genitourin$ or ureter$ or ureth$ or urin$ or urolog$ or urogen$ or catheter) adj5 (infect$ or bacteria$ or microbiol$ or abcess$)).tw. or (urin$ adj5 (abnormal$ or obstruct$ or fistula$ or calcul$ or stricture$)).tw. | 293375 |
| 2 | (amikacin$ or amoxicillin$ or ampicillin$ or aztreonam$ or cefepime$ or (cefiderocol$ or S-649266 or GSK-2696266) or cefoperazone$ or cefotaxime$ or ceftazidime$ or ceftibuten$ or ceftolozane$ or ceftriaxone$ or ciprofloxacin$ or colistin$ or colistimethate sodium or doripenem$ or (eravacycline$ or TP-434) or ertapenem$ or faropenem$ or fosfomycin$ or gentamicin$ or imipenem$ or levofloxacin$ or meropenem$ or minocycline$ or netilmicin$ or piperacillin$ or (plazomicin$ or ACHN-490) or polymixin$ or prulifloxacin$ or temocillin$ or ticarcillin$ or tobramycin$ or trimethoprim$ or (Relebactam$ or MK-7655)).tw. | 302966 |
| 3 | Clinical trial/ or Randomized controlled trial/ or Randomization/ or Single blind procedure/ or Double blind procedure/ or Crossover procedure/ or Placebo/ or Randomi?ed controlled trial$.tw. or Rct.tw. or Random allocation.tw. or Randomly allocated.tw. or Allocated | 4322791 |

| | | |
|---|---|---|
| | randomly.tw. or (allocated adj2 random).tw. or Single blind$.tw. or Double blind$.tw. or ((treble or triple) adj blind$).tw. or Placebo$.tw. or Prospective study/ or Randomized Controlled Trials as Topic/ or randomized controlled trial/ or Random Allocation/ or Double Blind Method/ or Single Blind Method/ or clinical trial/ or clinical trial, phase ii.pt. or clinical trial, phase iii.pt. or clinical trial, phase iv.pt. or controlled clinical trial.pt. or randomized controlled trial.pt. or multicenter study.pt. or clinical trial.pt. or exp Clinical Trials as topic/ or (clinical adj trial$).tw. or ((singl$ or doubl$ or treb$ or tripl$) adj (blind$3 or mask$3)).tw. or PLACEBOS/ or placebo$.tw. or randomly allocated.tw. or (allocated adj2 random$).tw. | |
| 4 | case report.tw. or letter/ or historical article/ or Case study/ or Case report.tw. or Abstract report/ or letter/ or Case study/ or Case report.tw. or Abstract report/ or letter/ | 4725669 |
| 5 | animal/ | 7817145 |
| 6 | human/ | 37234314 |
| 7 | 5 and 6 | 2233463 |
| 8 | 5 not 7 | 5583682 |
| 9 | 4 or 8 | 10218965 |
| 10 | 3 not 9 | 4073139 |
| 11 | 1 and 2 and 10 | 5209 |
| 12 | limit 11 to (english language and yr="2017 -Current") | 559 |
| 13 | remove duplicates from 12 | 435 |



Confidential - Not for Public Consumption or Distribution

## Table S15: Search strategy for COCHRANE LIBRARY (CENTRAL and CDSR) (1946 to May 17, 2019) for cUTI

| No | Search strings | Results |
|---|---|---|
| 1 | exp urinary tract infection/ or exp Pyelonephritis/ or exp Catheter-Related Infections/ or (urinary tract infection$ or uti or utis).tw. or pyelonephr$.tw. or bacteriuria$.tw. or (catheter$ adj2 infection$).tw. or ((genitourin$ or ureter$ or ureth$ or urin$ or urolog$ or urogen$ or catheter) adj5 (infect$ or bacteria$ or microbiol$ or abcess$)).tw. or (urin$ adj5 (abnormal$ or obstruct$ or fistula$ or calcul$ or stricture$)).tw. | 10994 |
| 2 | (amikacin$ or amoxicillin$ or ampicillin$ or aztreonam$ or cefepime$ or (cefiderocol$ or S-649266 or GSK-2696266) or cefoperazone$ or cefotaxime$ or ceftazidime$ or ceftibuten$ or ceftolozane$ or ceftriaxone$ or ciprofloxacin$ or colistin$ or colistimethate sodium or doripenem$ or (eravacycline$ or TP-434) or ertapenem$ or faropenem$ or fosfomycin$ or gentamicin$ or imipenem$ or levofloxacin$ or meropenem$ or minocycline$ or netilmicin$ or piperacillin$ or (plazomicin$ or ACHN-490) or polymixin$ or prulifloxacin$ or temocillin$ or ticarcillin$ or tobramycin$ or trimethoprim$ or (Relebactam$ or MK-7655)).tw. | 17452 |
| 3 | 1 and 2 | 2061 |
| 4 | limit 3 to english language [Limit not valid in CDSR; records were retained] | 1367 |
| 5 | limit 4 to yr="2017 -Current" | 136 |



Confidential - Not for Public Consumption or Distribution

**Table S16: Search strategy for MEDLINE (1946 to April 05, 2017) for cIAI**

| No | Search strings | Results |
|---|---|---|
| 1 | exp Intraabdominal Infections/ | 45651 |
| 2 | exp abdominal abscess/ | 11354 |
| 3 | (intra abdominal infection$ or intra-abdominal infection$ or intraabdominal infection$).tw. | 2207 |
| 4 | ((intraabdominal or intra-abdominal or abdominal) adj2 (infection$ or abscess$)).tw. | 6891 |
| 5 | appendicitis.tw. | 16467 |
| 6 | diverticulitis.tw. | 5539 |
| 7 | peritonitis.tw. | 27917 |
| 8 | or/1-7 | 77411 |
| 9 | amikacin$.tw. | 8198 |
| 10 | amoxicillin$.tw. | 12669 |
| 11 | ampicillin$.tw. | 20739 |
| 12 | aztreonam$.tw. | 2767 |
| 13 | cefepime$.tw. | 2695 |
| 14 | cefoperazone$.tw. | 2399 |
| 15 | cefotaxime$.tw. | 7543 |
| 16 | ceftazidime$.tw. | 7796 |
| 17 | ceftolozane$.tw. | 154 |
| 18 | ceftriaxone$.tw. | 8926 |
| 19 | cefuroxime$.tw. | 3939 |
| 20 | ciprofloxacin$.tw. | 21957 |
| 21 | colistin$.tw. | 3953 |
| 22 | colistimethate sodium$.tw. | 97 |
| 23 | doripenem$.tw. | 542 |
| 24 | (Eravacycline$ or TP-434).tw. | 40 |
| 25 | ertapenem$.tw. | 1147 |
| 26 | gentamicin$.tw. | 22259 |
| 27 | imipenem$.tw. | 9069 |
| 28 | levofloxacin$.tw. | 6199 |
| 29 | meropenem$.tw. | 4766 |
| 30 | moxifloxacin$.tw. | 3788 |
| 31 | piperacillin$.tw. | 5835 |
| 32 | polymixin$.tw. | 340 |
| 33 | ticarcillin$.tw. | 1936 |
| 34 | tigecycline$.tw. | 2363 |
| 35 | tobramycin$.tw. | 6289 |
| 36 | or/9-35 | 106855 |
| 37 | Randomized Controlled Trials as Topic/ | 111711 |
| 38 | randomized controlled trial/ | 457480 |
| 39 | Random Allocation/ | 91777 |



Confidential - Not for Public Consumption or Distribution

| No | Search strings | Results |
|---|---|---|
| 40 | Double Blind Method/ | 145956 |
| 41 | Single Blind Method/ | 24185 |
| 42 | clinical trial/ | 518663 |
| 43 | clinical trial, phase ii.pt. | 29780 |
| 44 | clinical trial, phase iii.pt. | 13503 |
| 45 | clinical trial, phase iv.pt. | 1443 |
| 46 | controlled clinical trial.pt. | 93386 |
| 47 | randomized controlled trial.pt. | 457480 |
| 48 | multicenter study.pt. | 223536 |
| 49 | clinical trial.pt. | 518663 |
| 50 | exp Clinical Trials as topic/ | 309936 |
| 51 | (clinical adj trial$).tw. | 294075 |
| 52 | ((singl$ or doubl$ or treb$ or tripl$) adj (blind$3 or mask$3)).tw. | 155976 |
| 53 | PLACEBOS/ | 34759 |
| 54 | placebo$.tw. | 193280 |
| 55 | randomly allocated.tw. | 22958 |
| 56 | (allocated adj2 random$).tw. | 25927 |
| 57 | or/37-56 | 1419984 |
| 58 | case report.tw. | 257176 |
| 59 | letter/ | 965438 |
| 60 | historical article/ | 344536 |
| 61 | animal/ | 6052916 |
| 62 | human/ | 16688414 |
| 63 | 61 and 62 | 1719309 |
| 64 | 61 not 63 | 4333607 |
| 65 | or/58-60,64 | 5842889 |
| 66 | 57 not 65 | 1307485 |
| 67 | 8 and 36 and 66 | 808 |
| 68 | limit 67 to english language | 711 |



Confidential - Not for Public Consumption or Distribution

## Table S17: Search strategy for EMBASE (1946 to April 05, 2017) for cIAI

| No | Searches | Results |
|---|---|---|
| 1 | exp abdominal infection/ | 21272 |
| 2 | exp appendicitis/ | 18088 |
| 3 | exp diverticulitis/ | 6367 |
| 4 | exp peritonitis/ | 39451 |
| 5 | (intra abdominal infection$ or intra-abdominal infection$ or intraabdominal infection$).tw. | 2800 |
| 6 | ((intraabdominal or intra-abdominal or abdominal) adj2 (infection$ or abscess$)).tw. | 8423 |
| 7 | appendicitis.tw. | 14297 |
| 8 | diverticulitis.tw. | 5947 |
| 9 | peritonitis.tw. | 27984 |
| 10 | or/1-9 | 80764 |
| 11 | amikacin$.tw. | 9476 |
| 12 | amoxicillin$.tw. | 17017 |
| 13 | ampicillin$.tw. | 18541 |
| 14 | aztreonam$.tw. | 3246 |
| 15 | cefepime$.tw. | 4188 |
| 16 | cefoperazone$.tw. | 2211 |
| 17 | cefotaxime$.tw. | 7728 |
| 18 | ceftazidime$.tw. | 9388 |
| 19 | ceftolozane$.tw. | 170 |
| 20 | ceftriaxone$.tw. | 12115 |
| 21 | cefuroxime$.tw. | 4509 |
| 22 | ciprofloxacin$.tw. | 28635 |
| 23 | colistin$.tw. | 4850 |
| 24 | colistimethate sodium$.tw. | 174 |
| 25 | doripenem$.tw. | 867 |
| 26 | (Eravacycline$ or TP-434).tw. | 80 |
| 27 | ertapenem$.tw. | 1851 |
| 28 | gentamicin$.tw. | 20271 |
| 29 | imipenem$.tw. | 12181 |
| 30 | levofloxacin$.tw. | 9645 |
| 31 | meropenem$.tw. | 7859 |
| 32 | moxifloxacin$.tw. | 5345 |
| 33 | piperacillin$.tw. | 7905 |
| 34 | polymixin$.tw. | 362 |
| 35 | ticarcillin$.tw. | 1505 |
| 36 | tigecycline$.tw. | 3566 |
| 37 | tobramycin$.tw. | 5822 |
| 38 | or/11-37 | 120068 |
| 39 | Clinical trial/ | 1004577 |



Confidential - Not for Public Consumption or Distribution

| No | Searches | Results |
|---|---|---|
| 40 | Randomized controlled trial/ | 471107 |
| 41 | Randomization/ | 83111 |
| 42 | Single blind procedure/ | 30582 |
| 43 | Double blind procedure/ | 133120 |
| 44 | Crossover procedure/ | 55916 |
| 45 | Placebo/ | 290718 |
| 46 | Randomi?ed controlled trial$.tw. | 157276 |
| 47 | Rct.tw. | 23689 |
| 48 | Random allocation.tw. | 1539 |
| 49 | Randomly allocated.tw. | 26054 |
| 50 | Allocated randomly.tw. | 2096 |
| 51 | (allocated adj2 random).tw. | 580 |
| 52 | Single blind$.tw. | 18010 |
| 53 | Double blind$.tw. | 154953 |
| 54 | ((treble or triple) adj blind$).tw. | 652 |
| 55 | Placebo$.tw. | 233802 |
| 56 | Prospective study/ | 401829 |
| 57 | or/39-56 | 1774206 |
| 58 | Case study/ | 95104 |
| 59 | Case report.tw. | 294676 |
| 60 | Abstract report/ or letter/ | 838379 |
| 61 | animal/ | 975357 |
| 62 | human/ | 15790687 |
| 63 | 61 and 62 | 283233 |
| 64 | 61 not 63 | 692124 |
| 65 | or/58-60,64 | 1902628 |
| 66 | 57 not 65 | 1694815 |
| 67 | 10 and 38 and 66 | 989 |
| 68 | limit 67 to english language | 874 |



Confidential - Not for Public Consumption or Distribution

**Table S18: Search strategy for COCHRANE LIBRARY [CENTRAL: November 2016 and CDSR: 2005 to January 25, 2017] for cIAI**

| No | Search strings | Results |
|---|---|---|
| 1 | (intra abdominal infection$ or intra-abdominal infection$ or intraabdominal infection$).tw. | 279 |
| 2 | (abdominal adj2 (infection$ or abscess$)).tw. | 700 |
| 3 | appendicitis.tw. | 649 |
| 4 | diverticulitis.tw. | 171 |
| 5 | peritonitis.tw. | 1072 |
| 6 | or/1-5 | 2384 |
| 7 | amikacin$.tw. | 650 |
| 8 | amoxicillin$.tw. | 2925 |
| 9 | ampicillin$.tw. | 1360 |
| 10 | aztreonam$.tw. | 279 |
| 11 | cefepime$.tw. | 201 |
| 12 | cefoperazone$.tw. | 230 |
| 13 | cefotaxime$.tw. | 662 |
| 14 | ceftazidime$.tw. | 780 |
| 15 | ceftolozane$.tw. | 13 |
| 16 | ceftriaxone$.tw. | 1045 |
| 17 | cefuroxime$.tw. | 728 |
| 18 | ciprofloxacin$.tw. | 1783 |
| 19 | colistin$.tw. | 187 |
| 20 | colistimethate sodium$.tw. | 20 |
| 21 | doripenem$.tw. | 36 |
| 22 | (Eravacycline$ or TP-434).tw. | 2 |
| 23 | ertapenem$.tw. | 83 |
| 24 | gentamicin$.tw. | 1444 |
| 25 | imipenem$.tw. | 454 |
| 26 | levofloxacin$.tw. | 831 |
| 27 | meropenem$.tw. | 271 |
| 28 | moxifloxacin$.tw. | 741 |
| 29 | piperacillin$.tw. | 596 |
| 30 | polymixin$.tw. | 15 |
| 31 | ticarcillin$.tw. | 239 |



Confidential - Not for Public Consumption or Distribution

| No | Search strings | Results |
|---|---|---|
| 32 | tigecycline$.tw. | 65 |
| 33 | tobramycin$.tw. | 1063 |
| 34 | (Relebactam$ or MK-7655).tw. | 0 |
| 35 | or/7-33 | 12181 |
| 36 | 6 and 35 | 574 |
| 37 | limit 36 to english language [Limit not valid in CDSR; records were retained] | 438 |



Confidential - Not for Public Consumption or Distribution

## Table S19: Search strategy for Embase (June 2017 to July 15, 2024)

| S. No. | Search strings | Results |
|---|---|---|
| 1 | exp Pneumonia/ | 370,865 |
| 2 | Respiratory Tract Infection/ | 56,843 |
| 3 | (respiratory adj3 infect$).tw. | 87,434 |
| 4 | (pneumon$ or bronchopneumon$ or pleuropneumon$ or bronchit$ or tracheobronchit$).tw. | 321,500 |
| 5 | or/1-4 | 586,304 |
| 6 | exp Cross Infection/ | 14,030 |
| 7 | cross infect$.tw. | 2,718 |
| 8 | (infection$ adj3 (hospital$ or nosocomial$ or health care$ or healthcare$ or health-care$)).tw. | 70,926 |
| 9 | ((hospital$ or nosocomial$ or ventilat$ or health care$ or healthcare$ or health-care$ or icu or intensive care) adj3 (acquired or associat$)).tw. | 127,975 |
| 10 | exp Mechanical ventilator/ | 7,404 |
| 11 | or/6-10 | 188,994 |
| 12 | 5 and 11 | 41,515 |
| 13 | exp hospital acquired pneumonia/ | 4,537 |
| 14 | exp health care associated pneumonia/ | 15,936 |
| 15 | exp ventilator associated pneumonia/ | 14,653 |
| 16 | (pneumon$ adj3 (hospital$ or nosocomial or ventilat$ or health care$ or health-care$ or healthcare$)).tw. | 27,129 |
| 17 | (HAP or VAP or HABP or VABP or HCAP).tw. | 18,236 |
| 18 | or/12-17 | 67,165 |
| 19 | amikacin$.tw. | 14,412 |
| 20 | aztreonam$.tw. | 4,161 |
| 21 | cefepime$.tw. | 7,942 |
| 22 | (cefiderocol$ or S-649266 or GSK-2696266).tw. | 968 |
| 23 | cefoperazone$.tw. | 2,652 |
| 24 | ceftazidime$.tw. | 13,919 |
| 25 | ceftolozane$.tw. | 1,306 |
| 26 | ciprofloxacin$.tw. | 40,882 |
| 27 | colistin$.tw. | 12,209 |
| 28 | colistimethate sodium$.tw. | 313 |
| 29 | doripenem$.tw. | 1,209 |
| 30 | ertapenem$.tw. | 3,567 |
| 31 | fosfomycin$.tw. | 4,560 |
| 32 | gentamicin$.tw. | 26,616 |
| 33 | imipenem$.tw. | 16,513 |
| 34 | levofloxacin$.tw. | 16,948 |
| 35 | meropenem$.tw. | 17,099 |
| 36 | minocycline$.tw. | 9,603 |
| 37 | piperacillin$.tw. | 13,629 |



Confidential - Not for Public Consumption or Distribution

| S. No. | Search strings | **Results** |
|---|---|---|
| 38 | (plazomicin$ or ACHN-490).tw. | 318 |
| 39 | polymixin$.tw. | 386 |
| 40 | tobramycin$.tw. | 7,423 |
| 41 | or/19-40 | 138,816 |
| 42 | clinical Trial/ | 946,761 |
| 43 | randomized controlled trial/ | 777,526 |
| 44 | Randomization/ | 91,084 |
| 45 | single blind procedure/ | 53,458 |
| 46 | double blind procedure/ | 193,038 |
| 47 | crossover procedure/ | 73,715 |
| 48 | placebo/ | 360,453 |
| 49 | Randomi?ed controlled trial$.tw. | 343,543 |
| 50 | rct.tw. | 57,397 |
| 51 | Random allocation.tw. | 2,383 |
| 52 | Randomly allocated.tw. | 43,669 |
| 53 | Allocated randomly.tw. | 2,403 |
| 54 | (allocated adj2 random).tw. | 463 |
| 55 | Single blind$.tw. | 29,715 |
| 56 | Double blind$.tw. | 206,529 |
| 57 | ((treble or triple) adj blind$).tw. | 2,046 |
| 58 | placebo$.tw. | 330,388 |
| 59 | Prospective study/ | 900,507 |
| 60 | or/42-59 | 2,646,205 |
| 61 | case study/ | 98,571 |
| 62 | case report.tw. | 505,811 |
| 63 | abstract report/ or letter/ | 973,249 |
| 64 | (rat or rats or mouse or mice or swine or porcine or murine or sheep or lambs or pigs or piglets or rabbit or rabbits or cat or cats or dog or dogs or cattle or bovine or monkey or monkeys or trout or marmoset$).ti. and animal experiment/ | 876,810 |
| 65 | 'animal experiment'/ not ('human experiment'/ or 'human'/) | 1,825,511 |
| 66 | or/61-65 | 3,446,696 |
| 67 | (2017$ or 2018$ or 2019$ or 2020$ or 2021$ or 2022$ or 2023$ or 2024$).em. | 15,784,905 |
| 68 | ("2017" or "2018" or "2019" or "2020" or "2021" or "2022" or "2023" or "2024").yr. | 12,968,884 |
| 69 | 60 not 66 | 2,546,642 |
| 70 | 18 and 41 and 69 | 1,038 |
| 71 | 70 and (67 or 68) | 518 |
| 72 | limit 71 to english language | 502 |



Confidential - Not for Public Consumption or Distribution

**Table S20: Search strategy for MEDLINE (June 2017 to July 15, 2024)**

| S. No. | Search strings | Results |
|---|---|---|
| 1 | exp Pneumonia/ | 368,883 |
| 2 | Respiratory Tract Infections/ | 43,808 |
| 3 | (respiratory adj3 infect$).tw. | 59,873 |
| 4 | (pneumon$ or bronchopneumon$ or pleuropneumon$ or bronchit$ or tracheobronchit$).tw. | 231,839 |
| 5 | or/1-4 | 580,974 |
| 6 | exp Cross Infection/ | 65,919 |
| 7 | cross infect$.tw. | 2,845 |
| 8 | (infection$ adj3 (hospital$ or nosocomial$ or health care$ or healthcare$ or health-care$)).tw. | 44,857 |
| 9 | ((hospital$ or nosocomial$ or ventilat$ or health care$ or healthcare$ or health-care$ or icu or intensive care) adj3 (acquired or associat$)).tw. | 75,394 |
| 10 | exp Ventilators, Mechanical/ | 10,397 |
| 11 | or/6-10 | 153,620 |
| 12 | 5 and 11 | 32,579 |
| 13 | Pneumonia, Ventilator-Associated/ | 4,454 |
| 14 | (pneumon$ adj3 (hospital$ or nosocomial or ventilat$ or health care$ or health-care$ or healthcare$)).tw. | 15,264 |
| 15 | (HAP or VAP or HABP or VABP or HCAP).tw. | 11,096 |
| 16 | or/12-15 | 44,460 |
| 17 | amikacin$.tw. | 10,105 |
| 18 | aztreonam$.tw. | 3,386 |
| 19 | cefepime$.tw. | 3,718 |
| 20 | (cefiderocol$ or S-649266 or GSK-2696266).tw. | 490 |
| 21 | cefoperazone$.tw. | 2,679 |
| 22 | ceftazidime$.tw. | 10,104 |
| 23 | ceftolozane$.tw. | 659 |
| 24 | ciprofloxacin$.tw. | 28,241 |
| 25 | colistin$.tw. | 7,607 |
| 26 | colistimethate sodium$.tw. | 148 |
| 27 | doripenem$.tw. | 681 |
| 28 | ertapenem$.tw. | 1,750 |
| 29 | fosfomycin$.tw. | 3,452 |
| 30 | gentamicin$.tw. | 25,434 |
| 31 | imipenem$.tw. | 11,147 |
| 32 | levofloxacin$.tw. | 8,674 |
| 33 | meropenem$.tw. | 8,193 |
| 34 | minocycline$.tw. | 7,223 |
| 35 | piperacillin$.tw. | 7,678 |
| 36 | (plazomicin$ or ACHN-490).tw. | 165 |



Confidential - Not for Public Consumption or Distribution

| S. No. | Search strings | Results |
|---|---|---|
| 37 | polymixin$.tw. | 367 |
| 38 | tobramycin$.tw. | 7,220 |
| 39 | or/17-38 | 102,312 |
| 40 | Randomized Controlled Trials as Topic/ | 170,762 |
| 41 | randomized controlled trial/ | 614,721 |
| 42 | Random Allocation/ | 107,314 |
| 43 | Double Blind Method/ | 178,940 |
| 44 | Single Blind Method/ | 33,588 |
| 45 | clinical trial/ | 539,084 |
| 46 | clinical trial, phase ii.pt. | 41,426 |
| 47 | clinical trial, phase iii.pt. | 22,825 |
| 48 | clinical trial, phase iv.pt. | 2,510 |
| 49 | controlled clinical trial.pt. | 95,530 |
| 50 | randomized controlled trial.pt. | 614,721 |
| 51 | multicenter study.pt. | 348,247 |
| 52 | clinical trial.pt. | 539,084 |
| 53 | exp Clinical Trials as topic/ | 393,177 |
| 54 | (clinical adj trial$).tw. | 434,396 |
| 55 | ((singl$ or doubl$ or treb$ or tripl$) adj (blind$3 or mask$3)).tw. | 185,269 |
| 56 | PLACEBOS/ | 35,965 |
| 57 | placebo$.tw. | 232,806 |
| 58 | randomly allocated.tw. | 32,173 |
| 59 | (allocated adj2 random$).tw. | 35,663 |
| 60 | or/40-59 | 1,872,716 |
| 61 | case report.tw. | 303,637 |
| 62 | letter/ | 1,207,461 |
| 63 | historical article/ | 370,271 |
| 64 | exp animals/ | 27,257,239 |
| 65 | humans/ | 22,026,946 |
| 66 | 64 and 65 | 22,026,946 |
| 67 | 64 not 66 | 5,230,293 |
| 68 | or/61-63,67 | 7,038,880 |
| 69 | 60 not 68 | 1,728,061 |
| 70 | (2017$ or 2018$ or 2019$ or 2020$ or 2021$ or 2022$ or 2023$ or 2024$).ez,ep,dt. | 7,007,293 |
| 71 | ("2017" or "2018" or "2019" or "2020" or "2021" or "2022" or "2023" or "2024").yr. | 7,147,270 |
| 72 | 16 and 39 and 69 | 653 |
| 73 | 72 and (70 or 71) | 176 |
| 74 | limit 73 to english language | 174 |



Confidential - Not for Public Consumption or Distribution

## Table S21: Search strategy for Cochrane Database of Systematic Reviews (June 2017 to July 15, 2024)

| S. No. | Search strings | Results |
|---|---|---|
| 1 | pneumon*:ti,ab,kw | 25984 |
| 2 | (respiratory NEAR/3 infect*):ti,ab,kw | 14694 |
| 3 | (pneumon* OR bronchopneumon* OR pleuropneumon* OR bronchit* OR tracheobronchit*):ti,ab,kw | 29941 |
| 4 | 37-#3 | 41339 |
| 5 | (cross NEXT infect*):ti,ab,kw | 1801 |
| 6 | (infection* NEAR/3 (hospital* OR nosocomial* OR health*)):ti,ab,kw | 6281 |
| 7 | ((hospital* OR (health NEXT care*) OR healthcare* OR nosocomial* OR ventilator* OR icu OR "intensive care") NEAR/3 (acquired OR associat*)):ti,ab,kw | 7747 |
| 8 | "artificial respiration":ti,ab,kw | 61 |
| 9 | "artificial ventilation":ti,ab,kw | 8049 |
| 10 | (mechanical NEXT ventilat*):ti,ab,kw | 14361 |
| 11 | {Panel, #5-`#10} | 29856 |
| 12 | #4 AND #11 | 6129 |
| 13 | (pneumon* NEAR/3 (hospital* OR nosocomial OR ventilat* OR (health NEXT care*) OR healthcare*)):ti,ab,kw | 3726 |
| 14 | (HAP OR VAP OR HABP OR VABP OR HCAP):ti,ab,kw | 1693 |
| 15 | 25-#14 | 7409 |
| 16 | (amikacin* OR aztreonam*):ti,ab,kw | 1399 |
| 17 | (cefepime* OR cefiderocol* OR "S 649266" OR "GSK 2696266" OR cefoperazone* OR ceftazidime*):ti,ab,kw | 1703 |
| 18 | (ceftolozane* OR ciprofloxacin*):ti,ab,kw | 3069 |
| 19 | (colistin* OR (colistimethate NEXT sodium*)):ti,ab,kw | 605 |
| 20 | (doripenem* OR ertapenem* OR fosfomycin*):ti,ab,kw | 711 |
| 21 | (gentamicin* OR imipenem* OR levofloxacin* OR meropenem* OR minocycline*):ti,ab,kw | 6832 |
| 22 | (piperacillin* OR plazomicin* OR "ACHN 490" OR polymixin*):ti,ab,kw | 1117 |
| 23 | tobramycin*:ti,ab,kw | 1551 |
| 24 | {OR #16-#23} | 13438 |
| 25 | #15 AND #24 with Cochrane Library publication date Between Apr 2017 and Jun 2024, in Cochrane Reviews | 2 |



Confidential - Not for Public Consumption or Distribution

**Table S22: Search strategy for Cochrane Central Register of Controlled Trials (June 2017 to July 15, 2024)**

| S. No. | Search strings | Results |
|---|---|---|
| 1 | pneumon*:ti,ab,kw | 25984 |
| 2 | (respiratory NEAR/3 infect*):ti,ab,kw | 14694 |
| 3 | (pneumon* OR bronchopneumon* OR pleuropneumon* OR bronchit* OR tracheobronchit*):ti,ab,kw | 29941 |
| 4 | 37-#3 | 41339 |
| 5 | (cross NEXT infect*):ti,ab,kw | 1801 |
| 6 | (infection* NEAR/3 (hospital* OR nosocomial* OR health*)):ti,ab,kw | 6281 |
| 7 | ((hospital* OR (health NEXT care*) OR healthcare* OR nosocomial* OR ventilator* OR icu OR "intensive care") NEAR/3 (acquired OR associat*)):ti,ab,kw | 7747 |
| 8 | "artificial respiration":ti,ab,kw | 61 |
| 9 | "artificial ventilation":ti,ab,kw | 8049 |
| 10 | (mechanical NEXT ventilat*):ti,ab,kw | 14361 |
| 11 | {Panel, #5-`#10} | 29856 |
| 12 | #4 AND #11 | 6129 |
| 13 | (pneumon* NEAR/3 (hospital* OR nosocomial OR ventilat* OR (health NEXT care*) OR healthcare*)):ti,ab,kw | 3726 |
| 14 | (HAP OR VAP OR HABP OR VABP OR HCAP):ti,ab,kw | 1693 |
| 15 | 25-#14 | 7409 |
| 16 | (amikacin* OR aztreonam*):ti,ab,kw | 1399 |
| 17 | (cefepime* OR cefiderocol* OR "S 649266" OR "GSK 2696266" OR cefoperazone* OR ceftazidime*):ti,ab,kw | 1703 |
| 18 | (ceftolozane* OR ciprofloxacin*):ti,ab,kw | 3069 |
| 19 | (colistin* OR (colistimethate NEXT sodium*)):ti,ab,kw | 605 |
| 20 | (doripenem* OR ertapenem* OR fosfomycin*):ti,ab,kw | 711 |
| 21 | (gentamicin* OR imipenem* OR levofloxacin* OR meropenem* OR minocycline*):ti,ab,kw | 6832 |
| 22 | (piperacillin* OR plazomicin* OR "ACHN 490" OR polymixin*):ti,ab,kw | 1117 |
| 23 | tobramycin*:ti,ab,kw | 1551 |
| 24 | {OR #16-#23} | 13438 |
| 25 | #15 AND #24 with Publication Year from 2017 to 2024, in Trials | 304 |



Confidential - Not for Public Consumption or Distribution

## Table S23: Search strategy for MEDLINe (1946 to April 05, 2017)

| S. No. | Search strings | Results |
|---|---|---|
| 1 | exp Pneumonia/ | 83563 |
| 2 | Respiratory Tract Infections/ | 35072 |
| 3 | (respiratory adj3 infect$).tw. | 43410 |
| 4 | (pneumon$ or bronchopneumon$ or pleuropneumon$ or bronchit$ or tracheobronchit$).tw. | 182314 |
| 5 | or/1-4 | 261128 |
| 6 | exp Cross Infection/ | 53289 |
| 7 | cross infect$.tw. | 2403 |
| 8 | (infection$ adj3 (hospital$ or nosocomial$ or health care$ or healthcare$ or health-care$)).tw. | 31539 |
| 9 | ((hospital$ or nosocomial$ or ventilat$ or health care$ or healthcare$ or health-care$ or icu or intensive care) adj3 (acquired or associat$)).tw. | 46053 |
| 10 | exp Ventilators, Mechanical/ | 8608 |
| 11 | or/6-10 | 109525 |
| 12 | 5 and 11 | 18799 |
| 13 | Pneumonia, Ventilator-Associated/ | 2632 |
| 14 | (pneumon$ adj3 (hospital$ or nosocomial or ventilat$ or health care$ or health-care$ or healthcare$)).tw. | 11211 |
| 15 | (HAP or VAP or HABP or VABP or HCAP).tw. | 7673 |
| 16 | or/12-15 | 26893 |
| 17 | amikacin$.tw. | 8198 |
| 18 | aztreonam$.tw. | 2767 |
| 19 | cefepime$.tw. | 2695 |
| 20 | (cefiderocol$ or S-649266 or GSK-2696266).tw. | 10 |
| 21 | cefoperazone$.tw. | 2399 |
| 22 | ceftazidime$.tw. | 7796 |
| 23 | ceftolozane$.tw. | 154 |
| 24 | ciprofloxacin$.tw. | 21957 |
| 25 | colistin$.tw. | 3953 |
| 26 | colistimethate sodium.tw. | 97 |
| 27 | doripenem$.tw. | 542 |
| 28 | ertapenem$.tw. | 1147 |
| 29 | fosfomycin$.tw. | 2313 |
| 30 | gentamicin$.tw. | 22259 |
| 31 | imipenem$.tw. | 9069 |
| 32 | levofloxacin$.tw. | 6199 |
| 33 | meropenem$.tw. | 4766 |
| 34 | minocycline$.tw. | 5747 |
| 35 | piperacillin$.tw. | 5835 |
| 36 | (plazomicin$ or ACHN-490).tw. | 46 |
| 37 | polymixin$.tw. | 340 |
| 38 | tobramycin$.tw. | 6289 |



Confidential - Not for Public Consumption or Distribution

| | | |
|---|---|---|
| 39 | or/17-38 | 79200 |
| 40 | Randomized Controlled Trials as Topic/ | 111711 |
| 41 | randomized controlled trial/ | 457480 |
| 42 | Random Allocation/ | 91777 |
| 43 | Double Blind Method/ | 145956 |
| 44 | Single Blind Method/ | 24185 |
| 45 | clinical trial/ | 518663 |
| 46 | clinical trial, phase ii.pt. | 29780 |
| 47 | clinical trial, phase iii.pt. | 13503 |
| 48 | clinical trial, phase iv.pt. | 1443 |
| 49 | controlled clinical trial.pt. | 93386 |
| 50 | randomized controlled trial.pt. | 457480 |
| 51 | multicenter study.pt. | 223536 |
| 52 | clinical trial.pt. | 518663 |
| 53 | exp Clinical Trials as topic/ | 309936 |
| 54 | (clinical adj trial$).tw. | 294075 |
| 55 | ((singl$ or doubl$ or treb$ or tripl$) adj (blind$3 or mask$3)).tw. | 155976 |
| 56 | PLACEBOS/ | 34759 |
| 57 | placebo$.tw. | 193280 |
| 58 | randomly allocated.tw. | 22958 |
| 59 | (allocated adj2 random$).tw. | 25927 |
| 60 | or/40-59 | 1419984 |
| 61 | case report.tw. | 257176 |
| 62 | letter/ | 965438 |
| 63 | historical article/ | 344536 |
| 64 | animal/ | 6052916 |
| 65 | human/ | 16688414 |
| 66 | 64 and 65 | 1719309 |
| 67 | 64 not 66 | 4333607 |
| 68 | or/61-63,67 | 5842889 |
| 69 | 60 not 68 | 1307485 |
| 70 | 16 and 39 and 69 | 512 |
| 71 | limit 70 to english language | 451 |



Confidential - Not for Public Consumption or Distribution

## Table S24: Search strategy for Embase (1988 to 2017 Week 14)

| S. No. | Search strings | Results |
|---|---|---|
| 1 | exp pneumonia/ | 228860 |
| 2 | respiratory tract infection/ | 46064 |
| 3 | (respiratory adj3 infect$).tw. | 53143 |
| 4 | (pneumon$ or bronchopneumon$ or pleuropneumon$ or bronchit$ or tracheobronchit$).tw. | 204157 |
| 5 | or/1-4 | 366864 |
| 6 | exp Cross Infection/ | 14259 |
| 7 | cross infect$.tw. | 2055 |
| 8 | (infection$ adj3 (hospital$ or nosocomial$ or health care$ or healthcare$ or health-care$)).tw. | 41427 |
| 9 | ((hospital$ or health care$ or healthcare$ or health-care$ or nosocomial$ or ventilator$ or icu or intensive care) adj3 (acquired or associat$)).tw. | 62225 |
| 10 | exp mechanical ventilator/ | 2046 |
| 11 | or/6-10 | 101700 |
| 12 | 5 and 11 | 23304 |
| 13 | exp hospital acquired pneumonia/ | 1868 |
| 14 | exp health care associated pneumonia/ | 587 |
| 15 | exp ventilator associated pneumonia/ | 9064 |
| 16 | (pneumon$ adj3 (hospital$ or nosocomial or ventilat$ or health care$ or health-care$ or healthcare$)).tw. | 16463 |
| 17 | (HAP or VAP or HABP or VABP or HCAP).tw. | 10807 |
| 18 | or/12-17 | 38084 |
| 19 | amikacin$.tw. | 9476 |
| 20 | aztreonam$.tw. | 3246 |
| 21 | cefepime$.tw. | 4188 |
| 22 | (cefiderocol$ or S-649266 or GSK-2696266).tw. | 21 |
| 23 | cefoperazone$.tw. | 2211 |
| 24 | ceftazidime$.tw. | 9388 |
| 25 | ceftolozane$.tw. | 170 |
| 26 | ciprofloxacin$.tw. | 28635 |
| 27 | colistin$.tw. | 4850 |
| 28 | colistimethate sodium.tw. | 174 |
| 29 | doripenem$.tw. | 867 |
| 30 | ertapenem$.tw. | 1851 |
| 31 | fosfomycin$.tw. | 2510 |
| 32 | gentamicin$.tw. | 20271 |
| 33 | imipenem$.tw. | 12181 |
| 34 | levofloxacin$.tw. | 9645 |
| 35 | meropenem$.tw. | 7859 |
| 36 | minocycline$.tw. | 6834 |
| 37 | piperacillin$.tw. | 7905 |



Confidential - Not for Public Consumption or Distribution

| | | |
|---|---|---|
| 38 | (plazomicin$ or ACHN-490).tw. | 106 |
| 39 | polymixin$.tw. | 362 |
| 40 | tobramycin$.tw. | 5822 |
| 41 | or/19-40 | 91446 |
| 42 | Clinical trial/ | 1004577 |
| 43 | Randomized controlled trial/ | 471107 |
| 44 | Randomization/ | 83111 |
| 45 | Single blind procedure/ | 30582 |
| 46 | Double blind procedure/ | 133120 |
| 47 | Crossover procedure/ | 55916 |
| 48 | Placebo/ | 290718 |
| 49 | Randomi?ed controlled trial$.tw. | 157276 |
| 50 | Rct.tw. | 23689 |
| 51 | Random allocation.tw. | 1539 |
| 52 | Randomly allocated.tw. | 26054 |
| 53 | Allocated randomly.tw. | 2096 |
| 54 | (allocated adj2 random).tw. | 580 |
| 55 | Single blind$.tw. | 18010 |
| 56 | Double blind$.tw. | 154953 |
| 57 | ((treble or triple) adj blind$).tw. | 652 |
| 58 | Placebo$.tw. | 233802 |
| 59 | Prospective study/ | 401829 |
| 60 | or/42-59 | 1774206 |
| 61 | Case study/ | 95104 |
| 62 | Case report.tw. | 294676 |
| 63 | Abstract report/ or letter/ | 838379 |
| 64 | animal/ | 975357 |
| 65 | human/ | 15790687 |
| 66 | 64 and 65 | 283233 |
| 67 | 64 not 66 | 692124 |
| 68 | or/61-63,67 | 1902628 |
| 69 | 60 not 68 | 1694815 |
| 70 | 18 and 41 and 69 | 762 |
| 71 | limit 70 to english language | 676 |



Confidential - Not for Public Consumption or Distribution

**Table S25: Search strategy for Cochrane library (CENTRAL: November 2016 and CDSR: 2005 to April 4, 2017)**

| S. No. | Search strings | Results |
|---|---|---|
| 1 | pneumon$.tw. | 8186 |
| 2 | (respiratory adj3 infect$).tw. | 4774 |
| 3 | (pneumon$ or bronchopneumon$ or pleuropneumon$ or bronchit$ or tracheobronchit$).tw. | 10166 |
| 4 | or/1-3 | 13830 |
| 5 | cross infect$.tw. | 109 |
| 6 | (infection$ adj3 (hospital$ or nosocomial$ or health$)).tw. | 2688 |
| 7 | ((hospital$ or health care$ or healthcare$ or health-care$ or nosocomial$ or ventilator$ or icu or intensive care) adj3 (acquired or associat$)).tw. | 4461 |
| 8 | artificial respiration.tw. | 38 |
| 9 | artificial ventilation.tw. | 219 |
| 10 | mechanical ventilat$.tw. | 4763 |
| 11 | or/5-10 | 10780 |
| 12 | 4 and 11 | 2140 |
| 13 | (pneumon$ adj3 (hospital$ or nosocomial or ventilat$ or health care$ or health-care$ or healthcare$)).tw. | 1603 |
| 14 | (HAP or VAP or HABP or VABP or HCAP).tw. | 575 |
| 15 | or/12-14 | 2735 |
| 16 | amikacin$.tw. | 669 |
| 17 | aztreonam$.tw. | 288 |
| 18 | cefepime$.tw. | 208 |
| 19 | (cefiderocol$ or S-649266 or GSK-2696266).tw. | 2 |
| 20 | cefoperazone$.tw. | 233 |
| 21 | ceftazidime$.tw. | 794 |
| 22 | ceftolozane$.tw. | 19 |
| 23 | ciprofloxacin$.tw. | 1817 |
| 24 | colistin$.tw. | 209 |
| 25 | colistimethate sodium.tw. | 22 |
| 26 | doripenem$.tw. | 40 |
| 27 | ertapenem$.tw. | 85 |
| 28 | fosfomycin$.tw. | 134 |
| 29 | gentamicin$.tw. | 1474 |
| 30 | imipenem$.tw. | 463 |
| 31 | levofloxacin$.tw. | 871 |
| 32 | meropenem$.tw. | 294 |
| 33 | minocycline$.tw. | 585 |
| 34 | piperacillin$.tw. | 620 |
| 35 | (plazomicin$ or ACHN-490).tw. | 2 |
| 36 | polymixin$.tw. | 19 |
| 37 | tobramycin$.tw. | 1085 |



Confidential - Not for Public Consumption or Distribution

| | | |
|---|---|---|
| 38 | or/16-37 | 7539 |
| 39 | 15 and 38 | 391 |
| 40 | limit 39 to english language [Limit not valid in CDSR; records were retained] | 297 |

**Figure S1: Risk of bias summary assessment for cUTI and cIAI systematic literature review**

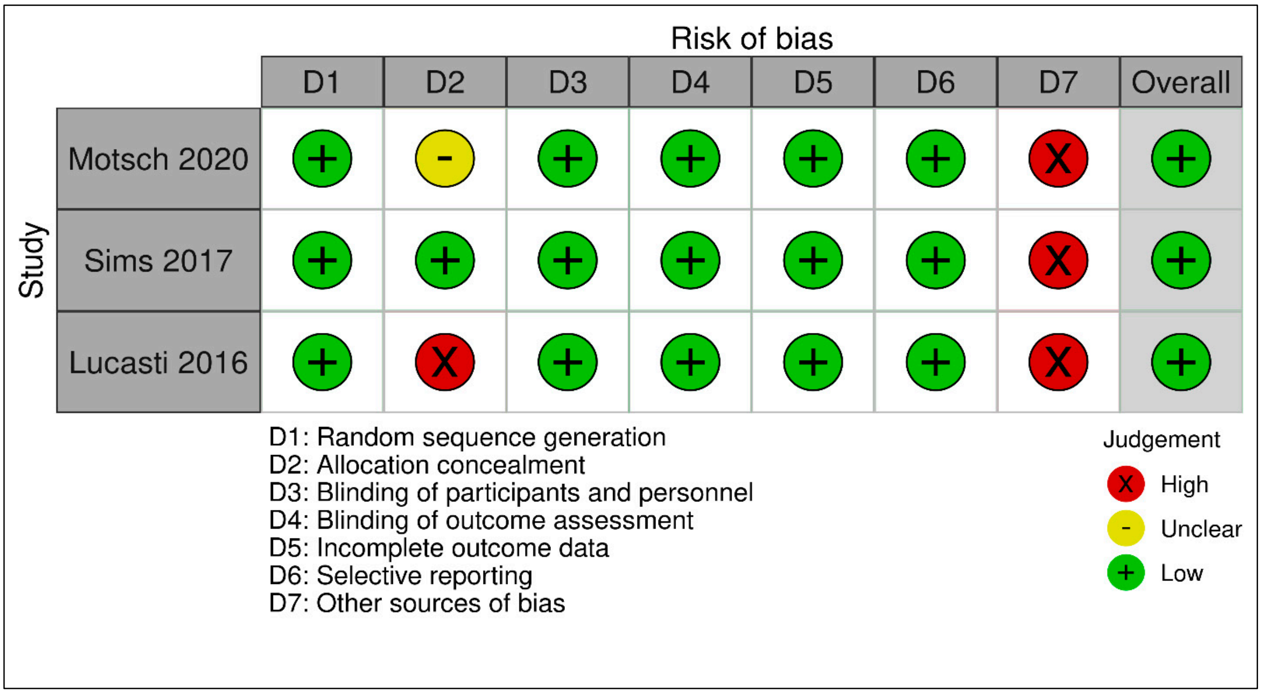




Confidential - Not for Public Consumption or Distribution

**Figure S2: Risk of bias summary assessment for HABP/VABP systematic literature review**

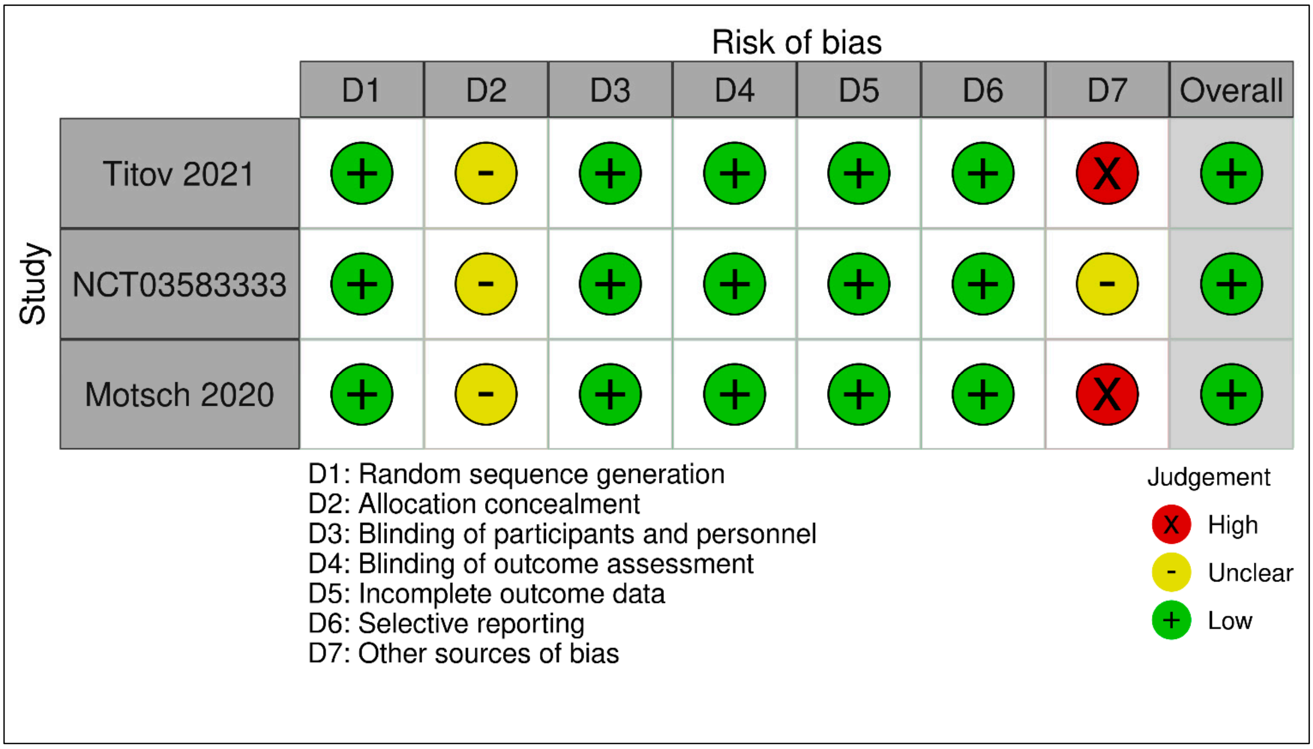




Confidential - Not for Public Consumption or Distribution
